# Transcriptional and Epigenetic Plasticity Drive an Alternative Non-clonal Mechanism of Resistance to *Kras*^G12D^ Inhibition in Pancreatic Cancer

**DOI:** 10.34133/cancomm.0030

**Published:** 2026-05-14

**Authors:** Alessia Caggiano, Antonio Agostini, Diego Iacuone, Lorenzo Priori, Annachiara Esposito, Anna Ceccarelli, Diego Rosa, Davide Pasini, Stefano Ugel, Francesco De Sanctis, Federica Cinti, Claudio Sette, Gian Luca Rampioni Vinciguerra, Clara Dezi, Vincenzo Bronte, Geny Piro, Vincenzo Corbo, Giampaolo Tortora, Carmine Carbone

**Affiliations:** ^1^ Department of Translational Medicine, Medical Oncology, Catholic University of the Sacred Heart, Rome 00168, Italy.; ^2^ Department of Medical and Surgical Sciences, Medical Oncology, Fondazione Policlinico Universitario Agostino Gemelli IRCCS, Rome 00168, Italy.; ^3^ Bioinformatics Research Core Facility, Gemelli Science and Technology Park (GSTeP), Fondazione Policlinico Universitario Agostino Gemelli IRCCS, Rome 00168, Italy.; ^4^ Department of Engineering for Innovation Medicine, University of Verona, Verona 37134, Italy.; ^5^ Department of Medicine, University of Verona, Verona 37129, Italy.; ^6^ Department of Medicine, Section of Immunology, University of Verona, Verona 37134, Italy.; ^7^ Scientific Directorate, Fondazione Policlinico Universitario Agostino Gemelli IRCCS, Rome 00168, Italy.; ^8^ Department of Neuroscience, Section of Human Anatomy, Catholic University of the Sacred Heart, Rome 00168, Italy.; ^9^ GSTeP-Organoids Research Core Facility, Fondazione Policlinico Universitario Agostino Gemelli IRCCS, Rome 00168, Italy.; ^10^ Department of Clinical and Molecular Medicine, Faculty of Medicine and Psychology, Sapienza University of Rome, Rome 00189, Italy.

Pancreatic ductal adenocarcinoma (PDAC) remains one of the most lethal malignancies, characterized by an aggressive clinical course and profound resistance to systemic therapies [1]. Oncogenic mutations in Kirsten rat sarcoma viral oncogene homolog (*KRAS*) are present in more than 90% of PDAC cases, with *KRAS^G12D^* representing the most frequent and clinically relevant allele [2]. The recent development of allele-specific noncovalent inhibitors targeting *KRAS^G12D^* represents a major breakthrough. Among these, MRTX1133 selectively binds *KRAS^G12D^*, locking it in its inactive guanosine diphosphate-bound state, and has shown strong antitumor activity in preclinical models, raising expectations for clinical translation [3]. However, emerging evidence indicates that therapeutic responses are limited by the rapid onset of resistance, whose mechanisms remain poorly understood [4]. While resistance is traditionally linked to clonal genetic evolution, including secondary mutations in drug target, bypass signaling pathways’ activation, or copy-number-driven oncogene amplification [5], these mechanisms do not fully explain the rapid and extensive resistance observed in PDAC. This suggests a role for additional nongenetic processes. Notably, PDAC exhibits high plasticity, with extensive transcriptional and phenotypic reprogramming [6]. We therefore hypothesized that resistance to KRAS^G12D^ inhibition may partly arise from adaptive, non-clonal mechanisms driven by transcriptional and epigenetic changes.

To investigate this hypothesis, we established in vivo models of acquired resistance to MRTX1133 by orthotopic inoculation of the KPC (*LSL-Kras^G12D/+^*; *LSL-Trp53^R172H/+^*; *Pdx1-Cre*) mouse-derived cell line FC1242, previously generated and characterized by our group [7,8], into syngeneic C57BL/6J mice, followed by treatment with increasing doses of MRTX1133 (1, 3, and 10 mg/kg; Supplementary Methods). Under sustained MRTX1133 high-dose treatment (10 mg/kg), tumors initially regressed but later resumed growth despite continued exposure, indicating acquired resistance (Fig. S1). Tumor cells from vehicle- and MRTX1133-treated mice were used to generate a nonresistant control (FC1242_2) and 2 first-generation resistant cell lines (Fig. 1A to C). Resistant cells maintained drug insensitivity upon reimplantation and long-term culture. Reimplantation of M1R_1 cells followed by treatment enabled derivation of second-generation resistant lines, namely, M2R_1 and M2R_2 (Fig. 1D to F). Dose–response analysis confirmed the reduced sensitivity of M1R and M2R cells compared to that of controls (Fig. S2A).

**Fig. 1.**
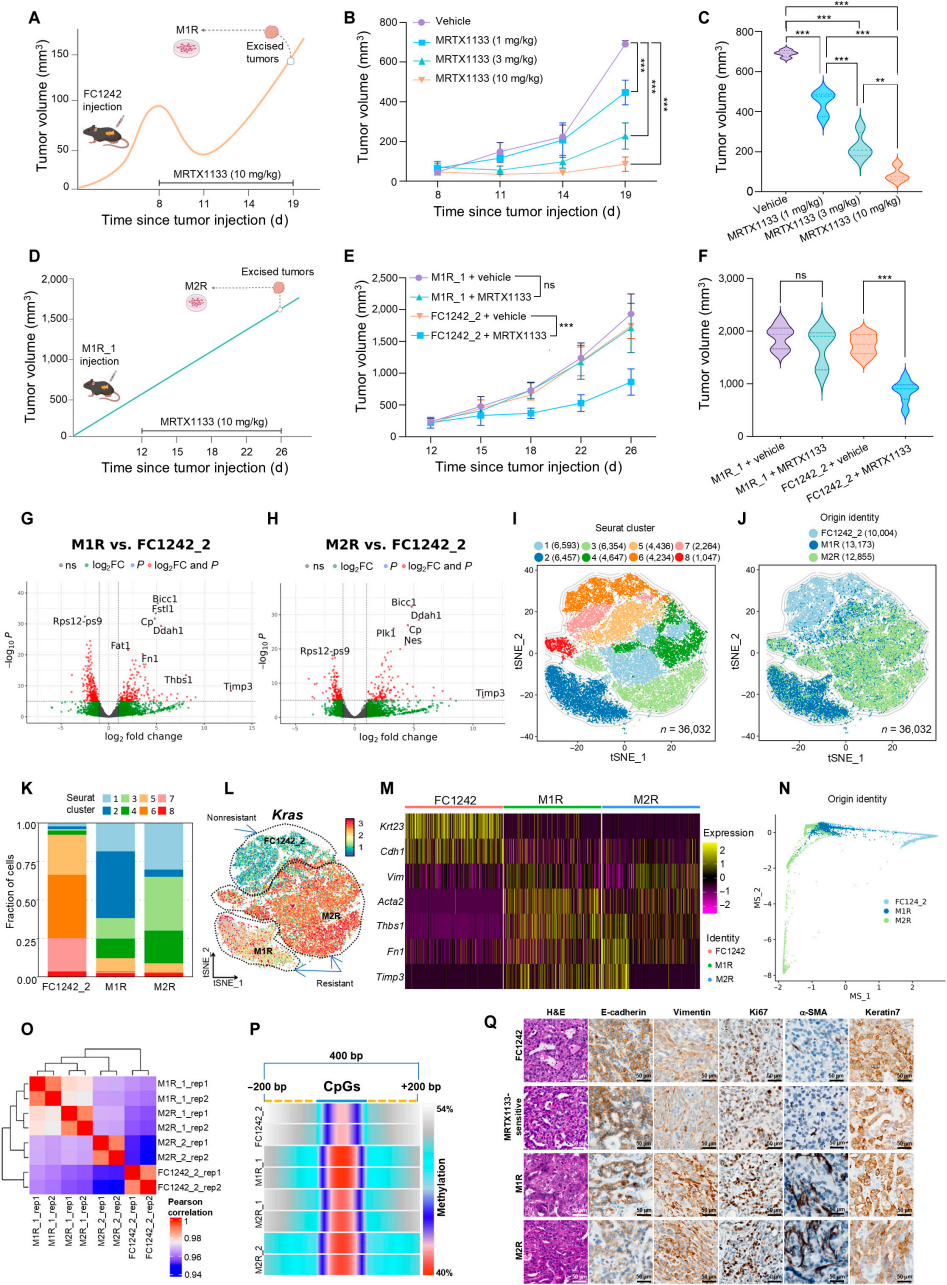
Adaptive transcriptional and epigenetic reprogramming drives resistance to *Kras^G12D^* inhibition in pancreatic cancer. (A) Schematic of the first in vivo experiment, showing the development of MRTX1133 resistance in mice inoculated with FC1242 cells and treated with a high dose of MRTX1133 (10 mg/kg), followed by the derivation of the first-generation MRTX1133-resistant cell line (M1R), namely, M1R_1 and M1R_2. (B) Tumor growth curves of C57BL/6J mice (*n* = 8 each group) orthotopically injected with the FC1242 pancreatic tumor cell line and treated with vehicle or increasing doses of MRTX1133 (1, 3, or 10 mg/kg, intraperitoneally, twice daily) for 11 d. The Jonckheere–Terpstra test confirmed a decreasing trend in tumor volume across increasing MRTX1133 dose groups (*P* < 0.001). (C) Endpoint (day 19 after tumor injection) tumor volume quantification of the first in vivo experiment showing comparison between all treatment groups. (D) Schematic illustrating the second in vivo experiment, showing the maintenance of MRTX1133 resistance in mice inoculated with M1R_1 cells treated with a high dose of MRTX1133 (10 mg/kg), followed by the derivation of the second-generation resistant cell line (M2R), namely, M2R_1 and M2R_2. (E) Tumor growth curves of the MRTX1133-resistant mouse model (C57BL/6J mice orthotopically injected with M1R_1 cells) and MRTX1133-sensitive mouse model (C57BL/6J mice orthotopically injected with the parental cell line FC1242) treated with vehicle or MRTX1133 (10 mg/kg, intraperitoneally, twice daily) for 14 d. Tumors in the MRTX1133-resistant group treated with MRTX1133 exhibited comparable tumor growth to those in the vehicle-treated MRTX1133-resistant group (*P* = 0.558), confirming stable resistance in vitro, whereas tumors in the MRTX1133-sensitive group treated with MRTX1133 showed a significantly slower tumor growth rate compared to those in the vehicle-treated MRTX1133-sensitive group (*P* < 0.001). (F) Endpoint (day 26 after tumor injection) tumor volume quantification of the second in vivo experiment showing comparison between all treatment groups. (G and H) Volcano plots from RNA sequencing analysis showing differentially expressed genes in M1R (integrated data with M1R_1 and M1R_2) (G) and M2R (integrated data with M2R_1 and M2R_2) (H) cell lines compared to nonresistant control FC1242_2 cells. DEA was performed using DESeq2 (significance thresholds were set at |log_2_ fold change| > 1, and −log_10_*P* > 5. (I and J) Plot showing t-SNE reduction of 36,032 single cells clustered using Seurat, showing the cells colored to 8 different clusters (I), and colored by sample origin: FC1242_2, M1R, and M2R (J). The cell counts of each subgroup are indicated in parentheses. (K) Stacked bar plot displaying the fraction of Seurat clusters across each cell line, showing the major cluster for each: FC1242_2 (clusters 5 to 7), M1R (cluster 2), and M2R (clusters 1, 3, and 4). Cluster 8 is conserved across all cell lines. (L) Expression of *Kras* displayed on the t-SNE map, indicating overexpression in MRTX1133-resistant cell clusters. (M) Heatmap of selected EMT markers’ expression (i.e., *Krt23*, *Vim*, *Cdh1*, *Acta2*, *Thbs1*, *Fn1*, and *Timp3*) across scRNA-seq samples, highlighting the overexpression of mesenchymal markers in MRTX1133-resistant cell lines. Expression levels are scaled and color coded (yellow: high expression; purple: low expression). (N) Palantir MS dimension reduction showing the plasticity of the identified clusters: M1R (cluster 2) displayed a major pluripotent state, whereas FC1242_2 (clusters 5 to 7) and M2R (clusters 1, 3, and 4) were more differentiated. The Palantir algorithm was used to infer cell fates and plasticity using the SeuratExtend package. (O and P) The DNA methylation sequencing of cell lines (FC1242_2, M1R_1, M2R_1, and M2R_2) was analyzed using the nf-core Methyl-seq pipeline. Heatmap of the Pearson correlation of global DNA methylation revealing distinct epigenetic profiles across samples, where rep1 and rep2 indicate the different replicates of sample (O), and plot of global CpG methylation (200 bp before and after CpG island) showing a progressive demethylation trend characteristic of MRTX1133-resistant cell lines (P). (Q) Representative H&E staining and IHC analysis for epithelial (E-cadherin and keratin 7), mesenchymal (vimentin and α-SMA), and proliferative (Ki67) markers on tumor tissues from C57BL/6J mice inoculated with different cell lines and subjected to distinct treatment conditions: vehicle-treated mice injected with FC1242, vehicle-treated mice injected with FC1242_2 (MRTX1133-sensitive), MRTX1133-treated mice injected with FC1242 that developed resistance (M1R), and MRTX1133-treated mice injected with M1R_1 that developed resistance (M2R). IHC revealed a phenotypic shift toward a more mesenchymal state in MRTX1133-resistant tumors. Scale bar: 50 μm. Statistical considerations: Graphs (B), (C), (E), and (F) show mean ± SD. One-way ANOVA was performed for statistical analyses (B, C, E, and F), with *P* < 0.05 (*), *P* < 0.01 (**), and *P* < 0.001 (***). The illustration was created using BioRender (A and D). *Acta2*, alpha smooth muscle actin; ANOVA, analysis of variance; bp, base pairs; *Cdh1*, cadherin 1; CpG, cytosine–phosphate–guanine; DEA, differential expression analysis; DESeq2, differential expression sequencing 2; EMT, epithelial–mesenchymal transition; FC1242, parental murine pancreatic cancer cell line; FC1242_2, nonresistant control, MRTX1133-sensitive murine pancreatic cancer cell line (_2 = secondary tumor-derived cell line); *Fn1*, fibronectin 1; H&E, hematoxylin–eosin; IHC, immunohistochemistry; Ki67, proliferation marker; *Kras*, Kirsten rat sarcoma viral oncogene homolog; *Krt23*, keratin 23; M1R, MRTX1133 first resistance (first-generation MRTX1133-resistant cell line); M1R_1, M = MRTX1133, 1 = first generation, R = resistant, _1 = mouse 1; M1R_2, M = MRTX1133, 1 = first generation, R = resistant, _2 = mouse 2; M2R, MRTX1133 second resistance (second-generation MRTX1133-resistant cell line); M2R_1, M = MRTX1133, 2 = second generation, R = resistant, _1 = mouse 1; M2R_2, M = MRTX1133, 2 = second generation, R = resistant, _2 = mouse 2; MRTX1133, *KRAS^G12D^* inhibitor; MS, multiscale space; *n*, sample size; ns, not significant; *P*, probability value; scRNA-seq, single-cell RNA sequencing; SD, standard deviation; t-SNE, t-distributed stochastic neighbor embedding; *Thbs1*, thrombospondin 1; *Timp3*, tissue inhibitor of metalloproteinase 3; *Vim*, vimentin.

Resistant cells showed comparable proliferation but increased motility relative to FC1242_2 (Fig. S2B and C), without cross-resistance to standard chemotherapies (Fig. S2D). MRTX1133 reduces KRAS pathway activation in sensitive models while showing no substantial effect in resistant ones, where signaling appears relatively maintained despite treatment (Figs. S3 and S4A to C). Consistently, resistant cells exhibited a shift toward a mesenchymal phenotype with altered epithelial–mesenchymal transition (EMT) marker expression (Fig. S4D and E).

Genomic analyses showed no evidence of canonical resistance mechanisms: whole-genome sequencing and copy number profiling identified no recurrent secondary *Kras* mutations, oncogene amplifications, or extrachromosomal DNA (Figs. S5 and S6; Table S1), supporting a nongenetic mechanism of resistance [9].

Transcriptomic profiling revealed marked divergence between resistant and control cells, with enrichment of EMT-related pathways (Fig. 1G and H and Fig. S7). These findings were corroborated by single-cell RNA sequencing, which identified distinct transcriptional clusters corresponding to nonresistant control (clusters 5 to 7) and MRTX1133-resistant cell lines (clusters 1 to 4), with MRTX1133-resistant populations enriched for EMT-like states absent in nonresistant control cell lines (Fig. 1I to K and Figs. S8 and S9). Notably, resistant cells showed increased Kras expression (Fig. 1L), likely reflecting an adaptive response to sustained KRAS inhibition rather than persistent oncogene dependency [4–10].

MRTX1133-resistant populations showed coordinated up-regulation of mesenchymal markers with suppression of epithelial lineage markers, including keratin 23 (*Krt23*), E-cadherin (*Cdh1*), vimentin (*Vim*), alpha smooth muscle actin (α-SMA; *Acta2*), thrombospondin 1 (*Thbs1*), fibronectin 1 (*Fn1*), and tissue inhibitor of metalloproteinase 3 (*Timp3*; Fig. 1M). Trajectory and cell fate analyses indicated increased transcriptional plasticity rather than terminal differentiation, supporting adaptive state switching over irreversible lineage commitment (Fig. 1N and Fig. S10). Given the stability of these changes, we examined epigenetic remodeling. DNA methylation profiling revealed progressive global and locus-specific demethylation in MRTX1133-resistant cells, particularly at regulatory regions of mesenchymal genes such as *Acta2*, *Thbs1*, lymphocyte antigen 6 family member E (*Ly6e*), and fibroblast growth factor 13 (*Fgf13*; Fig. 1O and P and Figs. S11 and S12). These alterations paralleled transcriptional activation, suggesting that epigenetic rewiring contributes to stabilizing resistance-associated reprogramming. Importantly, these features were recapitulated in vivo. Resistant tumors displayed a shift from well-formed glandular structures to poorly differentiated, spindle-shaped morphology. Immunohistochemistry in C57BL/6J mice injected with FC1242 or FC1242_2 (vehicle-treated, sensitive), FC1242-derived resistant tumors (M1R), and M1R-derived tumors (M2R) confirmed the loss of E-cadherin and increased vimentin and α-SMA, indicating EMT-driven plasticity under physiological conditions. In contrast, Ki67 and keratin 7 showed no significant changes (Fig. 1Q and Fig. S13).

Collectively, our findings support a model in which *Kras*^G12D^ inhibition imposes selective pressure that promotes transcriptional and epigenetic plasticity, enabling PDAC cells to adapt and reduce dependence on oncogenic *Kras* signaling without the acquisition of additional driver mutations. This non-clonal mechanism of resistance challenges the prevailing genetically centered paradigm and suggests tumor cell plasticity as a critical barrier to durable *KRAS*-targeted therapy response. Our data further suggest that combination strategies targeting EMT programs or epigenetic regulators may enhance the depth and durability of *KRAS^G12D^* inhibitor responses while EMT-associated transcriptomic and epigenetic signatures may serve as biomarkers of resistance. These findings have limitations. Although we observe a strong association with transcriptional and epigenetic reprogramming, functional validation is still lacking. Moreover, results are primarily based on murine models and require confirmation in human systems. Finally, this study provides an initial mechanistic framework that warrants further investigation into the drivers and reversibility of these adaptive states. We employed syngeneic orthotopic PDAC models (KPC-derived cells injected into C57BL/6J mice), which offer synchronized tumor onset and experimental control, although they do not fully recapitulate the histopathological and stromal complexity of spontaneous KPC models.

## Ethical Approval

Animal studies were approved by the Italian Ministry of Health (permit no. 566/2024-PR) and conducted in accordance with institutional guidelines.

## Data Availability

Data are publicly available: whole-genome sequencing, methylation sequencing, and bulk RNA sequencing (SRA: PRJNA1268994) and single-cell RNA sequencing (GEO: GSE298318). Additional data are available upon request.
