# Supplementary material for: Transcriptional and Epigenetic Plasticity Drive an Alternative Non-clonal Mechanism of Resistance to KrasG12D Inhibition in Pancreatic Cancer
Source: Cancer Commun (Lond). 2026 May 14;46:0030. doi: 10.34133/cancomm.0030 (PMC13172730; doi:10.34133/cancomm.0030)
Supplement: Supplementary 1 — Supplementary Methods Figs. S1 to S13 Table S1 [file cancomm.0030.f1.pdf]

Supplementary Materials for

Transcriptional and epigenetic plasticity drive an alternative non-clonal mechanism of resistance to $KRAS^{G12D}$ inhibition in pancreatic cancer

Alessia Caggiano[1], Antonio Agostini[2,3], Diego Iacuone[1], Lorenzo Priori[1], Annachiara Esposito[1], Anna Ceccarelli[1], Diego Rosa[4,5], Davide Pasini[4,5], Stefano Ugel[6], Francesco De Sanctis[6], Federica Cinti[7], Claudio Sette[8,9], Gian Luca Rampioni Vinciguerra[10], Carla Dezi[10], Vincenzo Bronte[6], Geny Piro[2], Vincenzo Corbo[4], Giampaolo Tortora[2], Carmine Carbone[2*].

Affiliation list

[1] Department of Translational Medicine, Medical Oncology, Catholic University of the Sacred Heart, Rome 00168, Italy.

[2] Department of Medical and Surgical Sciences, Medical Oncology, Fondazione Policlinico Universitario Agostino Gemelli IRCCS, Rome 00168, Italy.

[3] Bioinformatics Research Core Facility, Gemelli Science and Technology Park (GSTeP), Fondazione Policlinico Universitario Agostino Gemelli IRCCS, Rome 00168, Italy.

[4] Department of Engineering for Innovation Medicine, University of Verona, Verona 37134, Italy.

[5] Department of Medicine, University of Verona, Verona 37129, Italy.

[6] Department of Medicine, Section of Immunology, University of Verona, Verona 37134, Italy.

[7] Scientific Directorate, Fondazione Policlinico Universitario Agostino Gemelli IRCCS, Rome 00168, Italy.

[8] Department of Neuroscience, Section of Human Anatomy, Catholic University of the Sacred Heart, Rome 00168, Italy.

[9] GSTeP-Organoids Research Core Facility, Fondazione Policlinico Universitario Agostino Gemelli IRCCS, Rome 00168, Italy.

[10] Department of Clinical and Molecular Medicine, Faculty of Medicine and Psychology, Sapienza University of Rome, Rome 00189, Italy.

***Corresponding author:**

Carmine Carbone

Department of Medical and Surgical Sciences, Medical Oncology, Fondazione Policlinico Universitario Agostino Gemelli IRCCS, Rome 00168, Italy.

Email: carmine.carbone@policlinicogemelli.it

Telephone: +39 0630155894

Supplementary Methods

1. Pancreatic ductal adenocarcinoma mouse cell lines

Murine pancreatic ductal adenocarcinoma (PDAC) cell line FC1242, derived from KPC (LSL-*Kras*$^{G12D/+}$; LSL-*Trp53*$^{R172H/+}$; and *Pdx1*-Cre) mice, was kindly provided by Dr. David Tuveson's laboratory at Cold Spring Harbor Laboratory (New York, USA), and Dr. Paola Cappello's laboratory, at CeRMS (Centro di Ricerca in Medicina Sperimentale) laboratory (Turin, Italy), and passaged under original growth conditions. FC1242_2, M1R_1, M1R_2, M2R_1, and M2R_2 cell lines were obtained from excised tumors, as described in the "*in vivo* experiments" section below. Cells were inspected daily and routinely tested to be mycoplasma-free by PCR assay. MRTX1133-resistant cells were maintained *in vitro* under continuous administration of MRTX1133 (640 nmol/L).

2. Cell proliferation

In total, 1.0×10^3 cells/well were seeded in 96-well plates. For dose–response curves, non-resistant and MRTX1133-resistant cell lines were treated with standard chemotherapeutic agents, including GEM, ABX, and FOLFIRINOX (SN-38, OXA, and 5-FU), in the presence or absence of MRTX1133 (640 nmol/L) for 48 hours. Chemotherapeutic doses were selected based on their IC_{50} values. The concentration of MRTX1133 (640 nmol/L) was chosen as it corresponds to the dose used for continuous culture of resistant cells, approximately twice the IC_{50} of the non-resistant control line, allowing maintenance of selection pressure while preserving cell viability in resistant lines. At the indicated hours, sulforhodamine B (SRB; Sigma-Aldrich, Catalog No. S1402) assay was used to obtain relative estimates of viable cell numbers according to the manufacturer's instructions.

3. Wound healing assay

A wound healing assay was performed to evaluate the different migratory capacities of the established cell lines. Briefly, non-resistant control and resistant cells were cultured until they reached full confluence, and then they were maintained in 3% fetal bovine serum (FBS) Dulbecco's Modified Eagle Medium (DMEM). The wound was formed with a pipette, and images were captured at different time points for 24 hours. Images were acquired using the EVOS FL Auto 2 Cell Imaging System (Thermo Fisher Scientific, Catalog No. AMAFD2000) and analyzed with an ImageJ plugin to estimate the percentage of wound healing closure [1].

4. Protein extraction and Western blotting analyses

Non-resistant control and MRTX1133-resistant cell lines were treated with MRTX1133 (80 nmol/L) or vehicle for 6 hours, then the culture medium was aspirated and the cells were washed twice with ice-cold phosphate-buffered saline (PBS), scraped, and resuspended in RIPA buffer (Sigma-Aldrich, Catalog No. R0278) supplemented with Halt™ Protease and Phosphatase Inhibitor Cocktail (100×; Sigma-Aldrich, Catalog No. 78442). Tumor samples from *in vivo* experiments were mechanically minced using a sterile scalpel and subsequently homogenized in RIPA buffer containing protease and phosphatase inhibitors using VWR® Disposable Pellet Mixers and Cordless Motor (VWR International, Catalog No. 47747-370). Protein concentration was assessed using the Pierce™ BCA Protein Assay Kits (Thermo Fisher Scientific, Catalog No. 23227) following the manufacturer's instructions. Lysates were separated by sodium dodecyl sulfate–polyacrylamide gel electrophoresis (SDS-PAGE) and probed with the following antibodies from Cell Signaling Technology (CST): E-cadherin (Catalog No. 3195), Vimentin (Catalog No. 5741S), p44/42 mitogen-activated protein kinase (MAPK) (Erk1/2) (Catalog No. 4695), Phospho-p44/42 MAPK (Erk1/2) (Thr202/Tyr204) (Catalog No. 9106), S6 ribosomal protein (Catalog No. 2217), Phospho-S6 ribosomal protein (Ser235/236) (Catalog No. 2211), β-actin (Catalog No. 4970); from Abcam: anti-alpha smooth

muscle actin (Catalog No. ab124964); from Sigma-Aldrich: Vinculin (Catalog No. V9131). Gel blots were imaged with UVITEC Digital Image Scanning System Alliance Q9 (UVITEC).

5. Immunohistochemistry (IHC) and Hematoxylin and Eosin (H&E) staining

Whole 5 µm sections of the murine tumor, formalin-fixed, paraffin-embedded (FFPE; $n = 3$ per condition) were deparaffinized two times with xylene for 10 minutes and rehydrated two times with ethanol 100% for 3 minutes, then with ethanol 95% for 3 minutes, ethanol 75% for 3 minutes, and H_2O for 10 minutes. For IHC analysis, Citrate Plus (10×) HIER Solution (ScyTek Laboratories, Catalog No. CPL500) was used for heat-induced antigen retrieval, and 0.1% IGEPAL (Sigma-Aldrich, Catalog No. I3021) in PBS was used for permeabilization. The following antibodies were used for IHC staining with established procedures from CST: E-Cadherin (Rabbit 1:400, Catalog No. 3195), Vimentin (Rabbit 1:200, Catalog No. 5741S); from Abcam: Ki67 (1:200, Catalog No. ab16667), Cytokeratin 7 (1:8000, Catalog No. ab181598); from Invitrogen: Alpha-Smooth Muscle Actin (1:200, Catalog No. 14-9760-82). Endogenous peroxidases were blocked by using Peroxide Block for Image Analysis (ScyTek Laboratories ref: ADA500) for 15 minutes. Antigen detection was performed using 1 mL of 3,3′-diaminobenzidine (DAB) substrate high contrast (ScyTek Laboratories ref: ACU250) and 50 µL of DAB chromogen (ScyTek Laboratories ref: ABC030).

H&E staining was performed according to standard protocols with Mayer's Hematoxylin (ScyTek Laboratories ref: HMM500) and Bluing Reagent (ScyTek Laboratories ref: BRT500, Catalog No. NC1872006). The dehydration process was performed using ethanol 75%, 95%, and 100%, and xylene for 1 minute, then the slides were closed with EuKitt Quick-hardening mounting medium (Sigma-Aldrich, Catalog No. 25608-33-7). Images were acquired with the Ocus®40 Scanner (Grundium).

6. *In vivo* experiments

To generate syngeneic orthotopic PDAC cancer-derived graft (CDG) mouse models, female C57BL6/J recipient mice (4–6 weeks old) were injected with murine pancreatic cancer cells derived from KPC engineered mice that spontaneously develop PDAC. Cancer cells were resuspended in a 1:1 dilution of Matrigel (Corning Incorporated World, Corning, Catalog No. 356231) and cold PBS, and were injected into the tail region of the pancreas using insulin syringes (BD micro-fine 28 Gauge). The injection was considered successful by the development of a bubble without signs of leakage. The peritoneum was sutured with short-term absorbable suture (Vetsuture), and the skin was closed with wound clips. Tumor-bearing mice were subjected to high-contrast ultrasound screening twice a week using the Vevo 2100 System with an MS250, 13–24 MHz transducer (VisualSonics, Inc.). To evaluate *in vivo* the antitumor efficacy of MRTX1133 and to generate MRTX1133-resistant derivative cell lines, mice (n = 8, each group) were orthotopically inoculated with the FC1242 cell line (100,000 cancer cells per mouse) and randomly assigned to receive, via intraperitoneal injection twice daily for 11 days, vehicle, MRTX1133 (1 mg/kg), MRTX1133 (3 mg/kg) or MRTX1133 (10 mg/kg). Dosing was based on previously reported pharmacokinetic and pharmacodynamic data for MRTX1133 [2]. Tumor growth was monitored throughout the treatment period (11 days), and mice were euthanized at the end of the treatment for tumor inspection and tissue collection. Excised tumor tissues were mechanically dissociated and plated in 6-well plates to establish derivative cell lines. From vehicle-treated mice, a non-resistant control cell line (FC1242_2) was generated, while from resistant tumors specifically arising under high-dose (10 mg/kg) MRTX1133 treatment, the first-generation MRTX1133-resistant derivative cell lines (M1R) were established, namely M1R_1 and M1R_2. To verify if MRTX1133 resistance was maintained *in vitro*, mice (n = 8, each group) were inoculated with the M1R_1 cell line (250,000 cancer cells per mouse) and randomly assigned to receive, via intraperitoneal injection twice daily for 14

days, vehicle or MRTX1133 (10 mg/kg). Following the same procedure as above, second-generation MRTX1133-resistant cell lines (M2R) were successfully established and maintained in culture, namely M2R_1 and M2R_2. As a further control, another group of mice (*n* = 8 per group) was inoculated with the non-resistant control FC1242_2 cell line (MRTX1133-sensitive) and treated with the vehicle or the same high-dose regimen (MRTX1133, 10 mg/kg). These *in vivo* studies were designed to evaluate tumor response dynamics and investigate resistance mechanisms. All procedures were performed in accordance with ARRIVE 2.0 guidelines.

7. Genetic analysis

DNA was extracted from the non-resistant control FC1242_2 cell line and MRTX1133-resistant (M1R_1, M1R_2, M2R_1, and M2R_2) cell lines with the DNeasy Blood & Tissue Kit (Qiagen, Catalog No. 69504). Library preparation for whole-genome sequencing (WGS) was performed using the Illumina DNA Prep Kit (Illumina, Catalog No. 20060060), and sequencing was carried out by a certified external provider (NEGEDIA, Pozzuoli, NA, Italy, https://negedia.com/)on an Illumina NovaSeq 6000 system, producing paired-end 150 bp reads. Sequencing data were provided as demultiplexed FASTQ files and processed for quality control and downstream analyses. The nf-core/circdna (v1.1; https://github.com/nf-core/circdna) pipeline branch 'AmpliconArchitect' (AA) was used to align reads to the reference mm10 genome and to define amplicon classes in each sample. The nf-core/circdna calls copy number using cnvkit (v0.9.10) and prepares amplified segments with a copy number greater than 4.5 for AA by utilizing the functionality of the AmpliconSuite-Pipeline. AA (v1.3.r7) was run on the aligned reads and the amplified seeds to delineate the amplicon structures [3]. Identified amplicons were then classified using AmpliconClassifier (v1.1.1; https://github.com/AmpliconSuite/AmpliconClassifier) into circular extrachromosomal DNA

(ecDNA), linear amplicon, complex amplicon, or amplicon with a breakage-fusion-bridge signature (BFB) [4].

8. RNA sequencing (RNA-seq)

RNA was extracted from non-resistant control FC1242_2 cell line and MRTX1133-resistant (M1R_1, M1R_2, M2R_1, and M2R_2) cell lines with miRNeasy Micro Kit (Qiagen, Catalog No. 217084) to perform transcriptome sequencing (3' mRNA-Seq) with QuantSeq 3' mRNA-Seq V2 Library Prep Kit REV (Lexogen, Catalog No. 225.24). FASTQ files were processed and aligned with the Lexogen QuantSeq pipeline designed for 3′ mRNA sequencing (https://www.lexogen.com/quantseq-data-analysis/#:~:text=Analyze%20your%20QuantSeq%20data%20on%20your%20favourite%20data%20analysis%20platform). Briefly, raw reads were subjected to quality control and adapter trimming, followed by alignment to the reference genome GRCm38, and gene-level read counts were produced. The resulting count matrix was then used for downstream differential expression analysis (DEA) with DESeq2 [5]. Gene set enrichment analysis (GSEA) was performed with the R package clusterProfiler [6] to get insight into the biological processes modulated by the different treatments using the Mouse Molecular Signatures Database (MSigDB) Hallmark gene set (https://www.gsea-msigdb.org/gsea/msigdb/mouse/collections.jsp).

9. Single-cell RNA sequencing (scRNA-seq)

scRNA-seq was conducted in non-resistant control FC1242_2 cell line and MRTX1133-resistant cell lines (M1R_1, M2R_1, and M2R_2) with an average cell viability of 95%. scRNA-seq was performed with Chromium NEXT GEM Single Cell 5' kit (v2 - Dual Index) (10x Genomics, Catalog No. PN-1000265) according to the manufacturer's instructions. The

scRNA-seq library was sequenced on Novaseq 6000 (Illumina) following the manufacturer's recommendations at NEGEDIA S.r.l. (Pozzuoli, NA, Italy). FASTQ files were processed with Cell Ranger (v9.0.1; 10x Genomics), and outputs were analyzed with Seurat (v.5.2.1) [7]. Raw data were filtered, excluding cells with count percentages above 8% and 15% of mitochondrial and ribosomal genes, respectively. Counts were normalized and scaled, and embeddings were integrated with Harmony [8]. We got the curated gene set from Hwang single cell signature study [13]. We calculate the module scores for feature expression programs in single cells using the "AddModuleScore" function of Seurat. This function calculates the average expression levels of each program (cluster) on single cell level, subtracted by the aggregated expression of control feature sets. The Palantir algorithm was used to infer cell fates and plasticity using the SeuratExtend package (v.1.2.7) [9-10]. Given scRNA-seq data from a sample of differentiating cells and the expression profile of a user-defined 'early' cell, Palantir orders cells along a pseudotime, characterizes terminal differentiated states, and assigns each cell a probability distribution representing the cell's branch probability for reaching each terminal state.

10. Methylation sequencing (Methyl-seq)

DNA was extracted from FC1242_2, M1R_1, M2R_1, and M2R_2 with DNeasy Blood & Tissue Kit (Qiagen, Catalog No. 69504). Samples were processed with NEBNext Enzymatic Methyl-seq v2 Kit (New England Biolabs, Catalog No. E8015) and sequenced with DNBSEQ-T7 (Mouse Genome Informatics) at NEGEDIA S.r.l. FASTQ files were analyzed with the nf-core [11] pipeline Methylseq (https://zenodo.org/records/17910147), consisting of the combination of Bowtie 2 and Bismarck for alignment and methylation extraction. The Bismark coverage file was imported on SeqMonk (https://www.bioinformatics.babraham.ac.uk/projects/seqmonk/) for differential methylation analysis and visualization.

11. Statistical analysis

All results, where applicable, were expressed as the means ± standard deviation (SD). All statistical analyses were performed using GraphPad Prism version 9.0 (GraphPad Software). $P < 0.05$ was considered statistically significant, specifically $P < 0.05$ (*), $P < 0.01$ (**), and $P < 0.001$ (***). One-way analysis of variance (ANOVA) was applied to calculate the statistical significance between multiple group comparisons when data were normally distributed; otherwise, the Kruskal–Wallis test followed by Dunn's multiple comparisons test was applied.

References

1. Suarez-Arnedo A, Torres Figueroa F, Clavijo C, Arbeláez P, Cruz JC, Muñoz-Camargo C. An image J plugin for the high throughput image analysis of in vitro scratch wound healing assays. PLoS One. 2020;15(7):e0232565.
2. Kemp SB, Cheng N, Markosyan N, Sor R, Kim IK, Hallin J, et al. Efficacy of a Small-Molecule Inhibitor of KrasG12D in Immunocompetent Models of Pancreatic Cancer. Cancer Discov. 2023;13(2):298–311.
3. Deshpande V, Luebeck J, Nguyen ND, Bakhtiari M, Turner KM, Schwab R, et al. Exploring the landscape of focal amplifications in cancer using AmpliconArchitect. Nat Commun. 2019;10(1):392.
4. Luebeck J, Ng AWT, Galipeau PC, Li X, Sanchez CA, Katz-Summercorn AC, et al. Extrachromosomal DNA in the cancerous transformation of Barrett's oesophagus. Nature. 2023;616(7958):798–805.
5. Love MI, Huber W, Anders S. Moderated estimation of fold change and dispersion for RNA-seq data with DESeq2. Genome Biol. 2014;15(12):550.
6. Yu G, Wang LG, Han Y, He QY. clusterProfiler: an R package for comparing biological themes among gene clusters. Omics. 2012;16(5):284–7.
7. Hao Y, Stuart T, Kowalski MH, Choudhary S, Hoffman P, Hartman A, et al. Dictionary learning for integrative, multimodal and scalable single-cell analysis. Nat Biotechnol. 2024;42(2):293–304.
8. Korsunsky I, Millard N, Fan J, Slowikowski K, Zhang F, Wei K, et al. Fast, sensitive and accurate integration of single-cell data with Harmony. Nat Methods. 2019;16(12):1289–96.
9. Hua Y, Weng L, Zhao F, Rambow F. SeuratExtend: streamlining single-cell RNA-seq analysis through an integrated and intuitive framework. Gigascience. 2025;14.
10. Setty M, Kiseliovas V, Levine J, Gayoso A, Mazutis L, Pe'er D. Characterization of cell fate probabilities in single-cell data with Palantir. Nat Biotechnol. 2019;37(4):451–60.

11. Ewels PA, Peltzer A, Fillinger S, Patel H, Alneberg J, Wilm A, et al. The nf-core framework for community-curated bioinformatics pipelines. Nat Biotechnol. 2020;38(3):276–8.
12. Luo J. KRAS mutation in pancreatic cancer. Semin Oncol. 2021 Feb;48(1):10-18.
13. Hwang WL, Jagadeesh KA, Guo JA, Hoffman HI, Yadollahpour P, Reeves JW, et al. Single-nucleus and spatial transcriptome profiling of pancreatic cancer identifies multicellular dynamics associated with neoadjuvant treatment. Nat Genet. 2022;54(8):1178–91.

Supplementary Figures

(A)

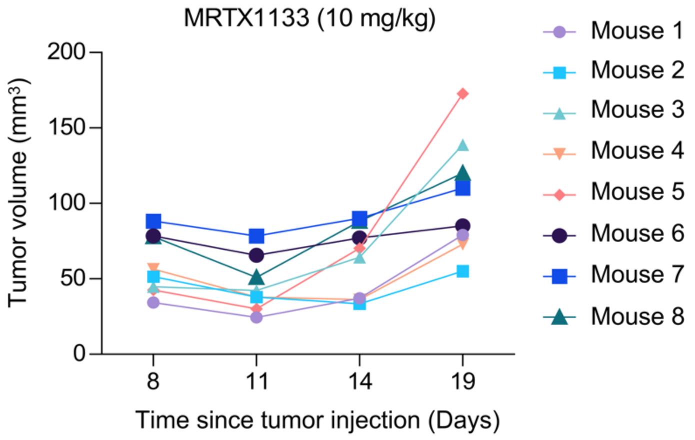

(B)

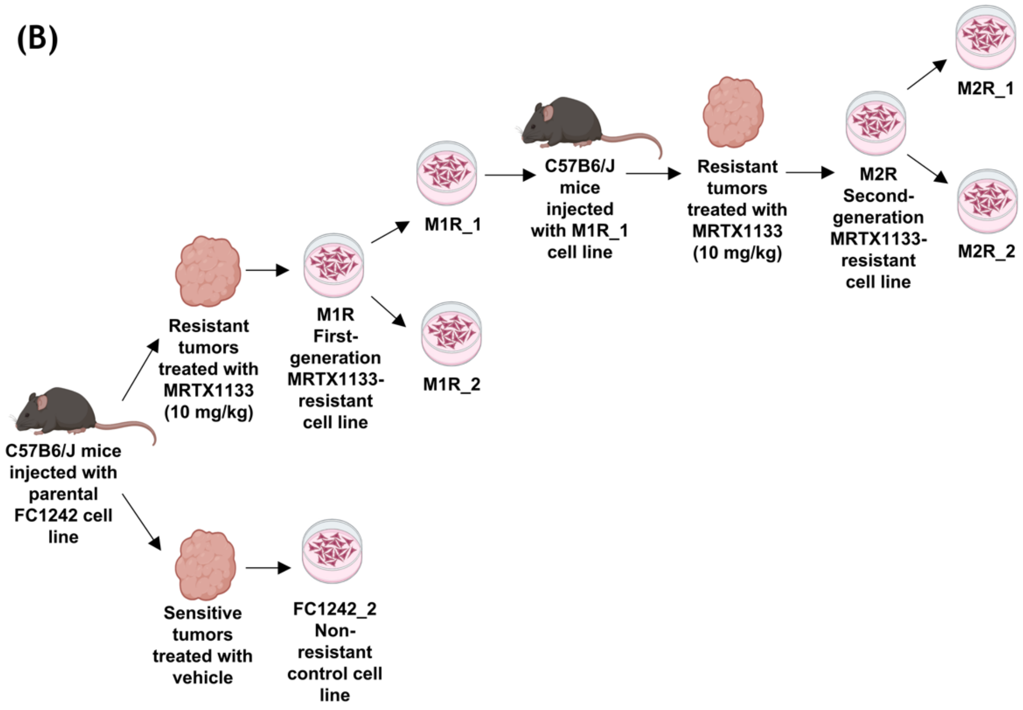

Supplementary Figure S1. Generation of *in vivo* MRTX1133-resistant models. (A) Tumor growth curves of C57BL/6J mice (*n* = 8) orthotopically injected with FC1242 pancreatic tumor cell line and treated with MRTX1133 (10 mg/kg, intraperitoneally, twice daily) for 11 days. Tumor growth was evaluated twice per week starting 8 days after tumor injection, when

MRTX1133 treatment was initiated. At day 11, a clear reduction in tumor size in all mice was observed, indicative of an initial therapeutic response. However, after an additional 3 days of continuous dosing, at day 14, tumor growth resumed and progressively increased until day 19, despite ongoing MRTX1133 administration. This regrowth phase was interpreted as the onset of acquired resistance. **(B)** Flow chart illustrating the development of resistance models. FC1242 cells were inoculated into C57BL/6J mice. Tumors obtained from vehicle-treated animals were used to generate the FC1242_2 cell line (FC1242_2 was named to indicate that it represents a secondary tumor-derived cell line obtained after one round of in vivo passage). Tumors arising under high-dose (10 mg/kg) MRTX1133 treatment were used to establish first-generation resistant cell lines (M1R). Two biological replicate cell lines (M1R_1 and M1R_2) were independently derived from two distinct mice. In this nomenclature, “M1R” indicates first-generation resistance to MRTX1133 (M = MRTX1133; 1 = first generation; R = resistant), while the suffix (_1 or _2) identifies the mouse of origin. M1R_1 cells were inoculated again into mice and treated with MRTX1133 (10 mg/kg). From tumors derived from M1R_1-inoculated, MRTX1133-treated mice, second-generation resistant cell lines (M2R) were established, namely M2R_1 and M2R_2. Here, “M2R” denotes second-generation resistance (M = MRTX1133; 2 = second generation; R = resistant), and the suffix (_1 or _2) refers to the individual mouse of origin.

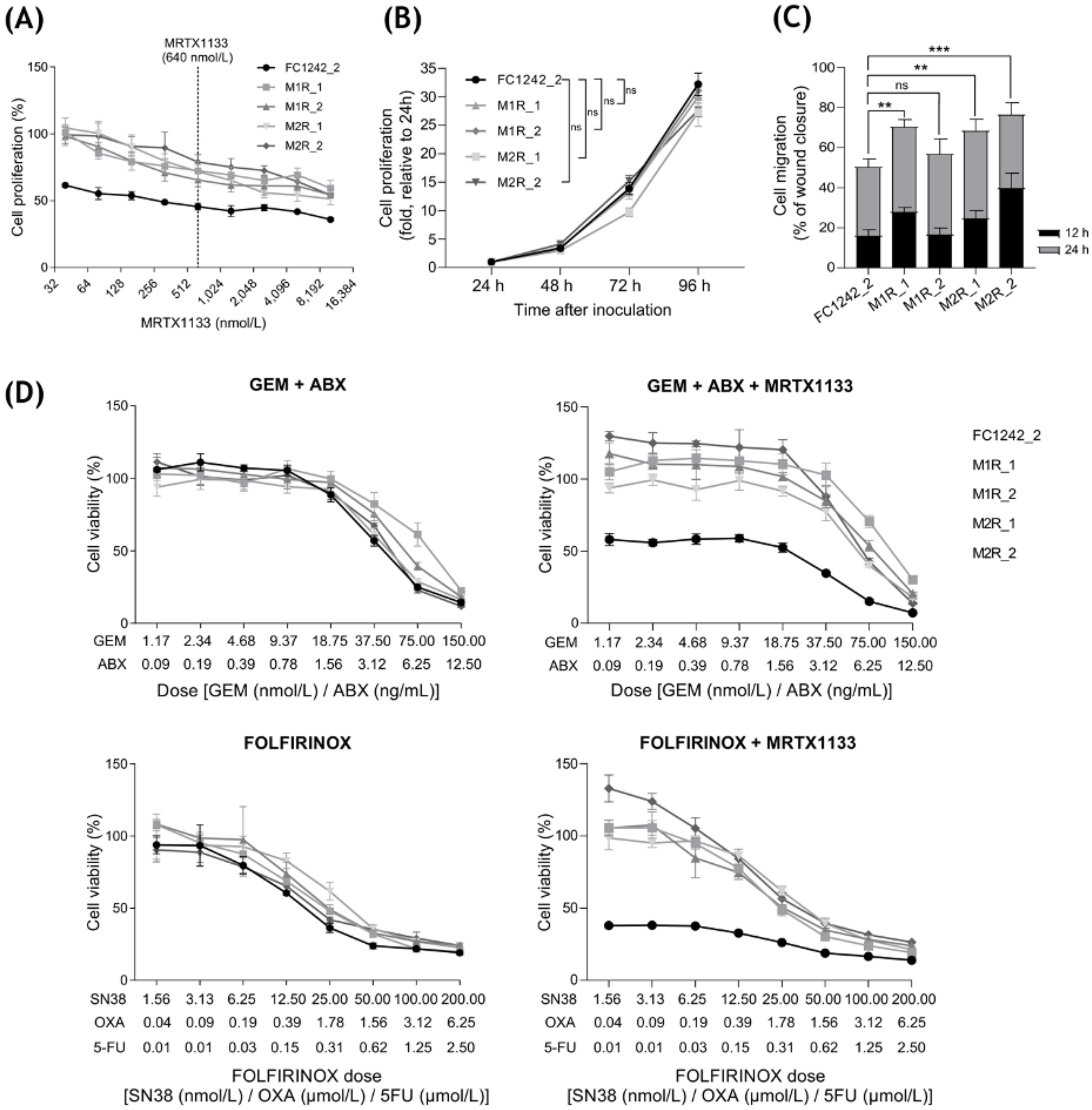

Supplementary Figure S2. Functional and molecular characterization of MRTX1133-resistant pancreatic cancer cell lines. (A) MRTX1133 dose–response curves of non-resistant control (FC1242_2) and MRTX1133-resistant (M1R_1, M1R_2, M2R_1, M2R_2) cell lines. Dose–response analysis revealed a significantly reduced sensitivity to MRTX1133 in MRTX1133-resistant cells compared with the non-resistant control (FC1242_2 *vs.* M1R_1, $P < 0.01$; FC1242_2 *vs.* M1R_2, $P < 0.05$; FC1242_2 *vs.* M2R_1, $P < 0.01$; FC1242_2 *vs.*

M2R_2, P < 0.001). **(B)** Cell proliferation of non-resistant control (FC1242_2) and MRTX1133-resistant (M1R_1, M1R_2, M2R_1, M2R_2) cell lines cultured over 96 hours. Resistant cells were cultured under chronic drug exposure conditions. Proliferation is expressed as a fold change relative to 24 hours. **(C)** Quantification of cell migration (percentage of wound closure) at 12 and 24 hours after wound creation. Statistical analysis was performed on the 24 h time point. **(D)** Dose–response curves of parental (FC1242_2) and MRTX1133-resistant (M1R_1, M1R_2, M2R_1, M2R_2) cell lines treated with GEM + ABX ± MRTX1133 (640 nmol/L) and FOLFIRINOX (SN-38 + OXA + 5-FU) ± MRTX1133 (640 nmol/L) for 48 hours. 640 nmol/L corresponds to approximately twice the IC_{50} of the parental cells (FC1242_2) and was chosen to maintain selective pressure while preserving cell viability in the resistant lines. Graphs (A, B, C and D) show the mean ± SD. Statistical analysis (C) was performed using ordinary one-way ANOVA. Drug sensitivity (A) was quantified in dose-response analysis and statistical significance was assessed using the Kruskal–Wallis test followed by Dunn's multiple comparisons test. $P < 0.05$ (*), $P < 0.01$ (**), and $P < 0.001$ (***). Abbreviations: 5-FU, 5-fluorouracil; ABX, Abraxane; ANOVA, analysis of variance;; FC1242_2, parental MRTX1133-sensitive murine pancreatic cancer cell line; FOLFIRINOX, combined chemotherapy regimen including 5-FU, SN-38, and OXA; GEM, gemcitabine; GEM/ABX, combined chemotherapy regimen including GEM and ABX; M1R, MRTX1133 1st resistance (first-generation MRTX1133-resistant derivative cell line); M2R, MRTX1133 2nd resistance (second-generation MRTX1133-resistant derivative cell line); MRTX1133, $KRAS^{G12D}$ inhibitor; OXA, oxaliplatin; SD, standard deviation; SN-38, active metabolite of irinotecan

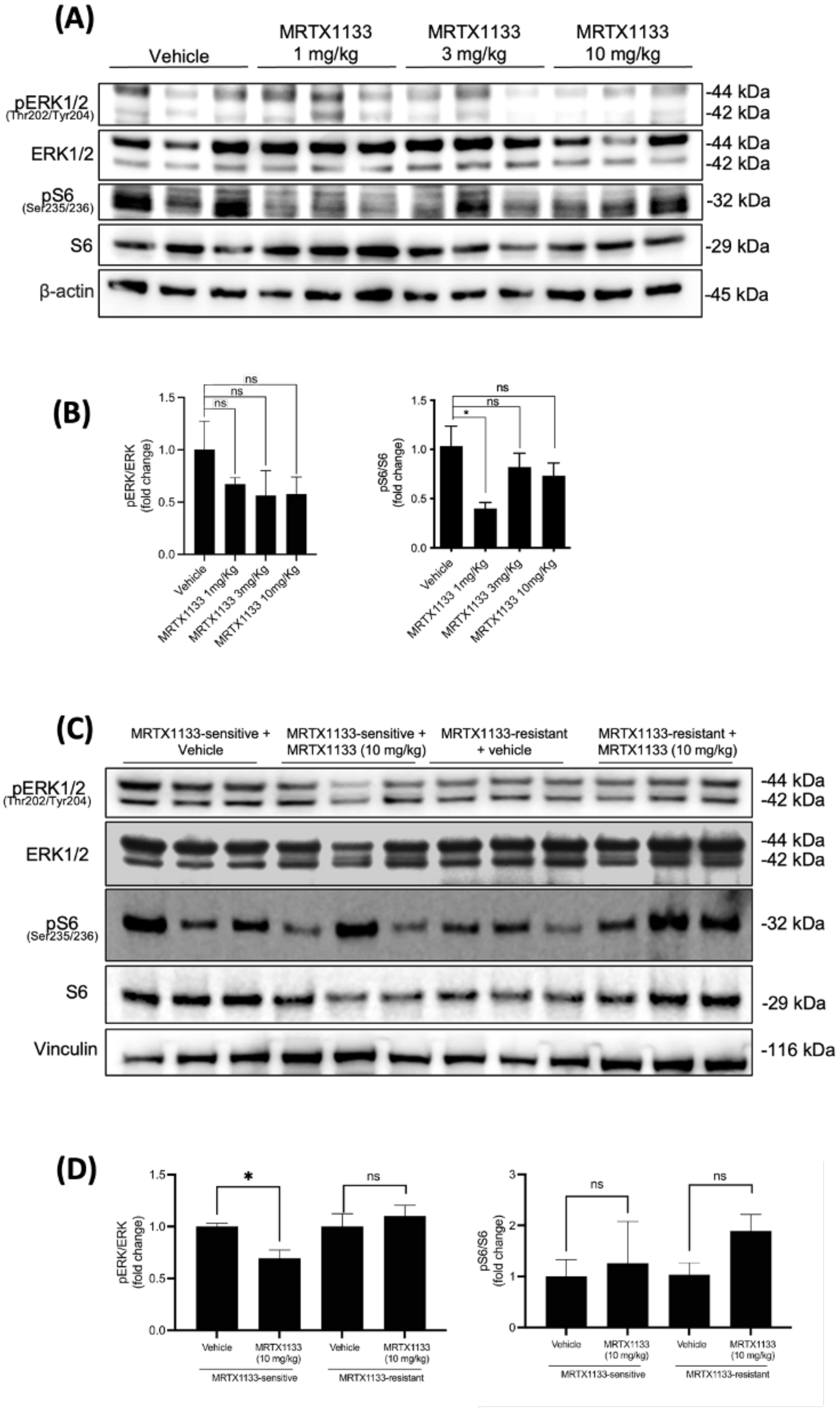

Supplementary Figure S3. ***In vivo*** **models recapitulate MRTX1133 resistance in pancreatic cancer** mouse model**. (A)** Western blotting analyses of the key markers of the best-

characterized and most functionally relevant *KRAS* downstream signaling pathways in PDAC mouse model, MAPK/ERK pathway [pERK1/2 (Thr202/Tyr204) and ERK1/2] and PI3K/AKT/mTOR pathway [pS6 (Ser235/236) and S6]. Tumor lysates analyzed were derived from the first *in vivo* experiment (*n* = 3 mice, each group), specifically from C57BL/6J mice inoculated with the parental FC1242 cell line and treated with vehicle or increasing doses of MRTX1133 (1, 3, or 10 mg/kg). The indicated ratios of phosphorylated to total protein are shown, normalized to vehicle-treated controls. **(B)** Densitometric quantification of the Western blotting results shown in Supplementary Figures S3A. **(C)** Western blotting analyses of the key markers of the best-characterized and most functionally relevant *KRAS* downstream signaling pathways in PDAC mouse model, MAPK/ERK [pERK1/2 (Thr202/Tyr204) and ERK1/2] and PI3K/AKT/mTOR [pS6 (Ser235/236) and S6]. Tumor lysates analyzed were derived from the second *in vivo* experiment (*n* = 3 mice, each group), specifically from C57BL/6J mice inoculated with FC1242_2 or M1R_1 and treated with vehicle or MRTX1133 (10 mg/kg). The indicated ratios of phosphorylated to total protein are shown, normalized to vehicle-treated controls. **(D)** Densitometric quantification of the Western blotting results shown in Supplementary Figures S3C. Abbreviations: AKT, protein kinase B; ERK1/2, extracellular signal-regulated kinases 1 and 2; MAPK, mitogen-activated protein kinase; mTOR, mechanistic target of rapamycin; *n*, sample size; p, phosphorylated; PI3K, phosphoinositide 3-kinase; PDAC, pancreatic ductal adenocarcinoma; S6, ribosomal protein S6; Ser, serine; Thr, threonine; Tyr, tyrosine.

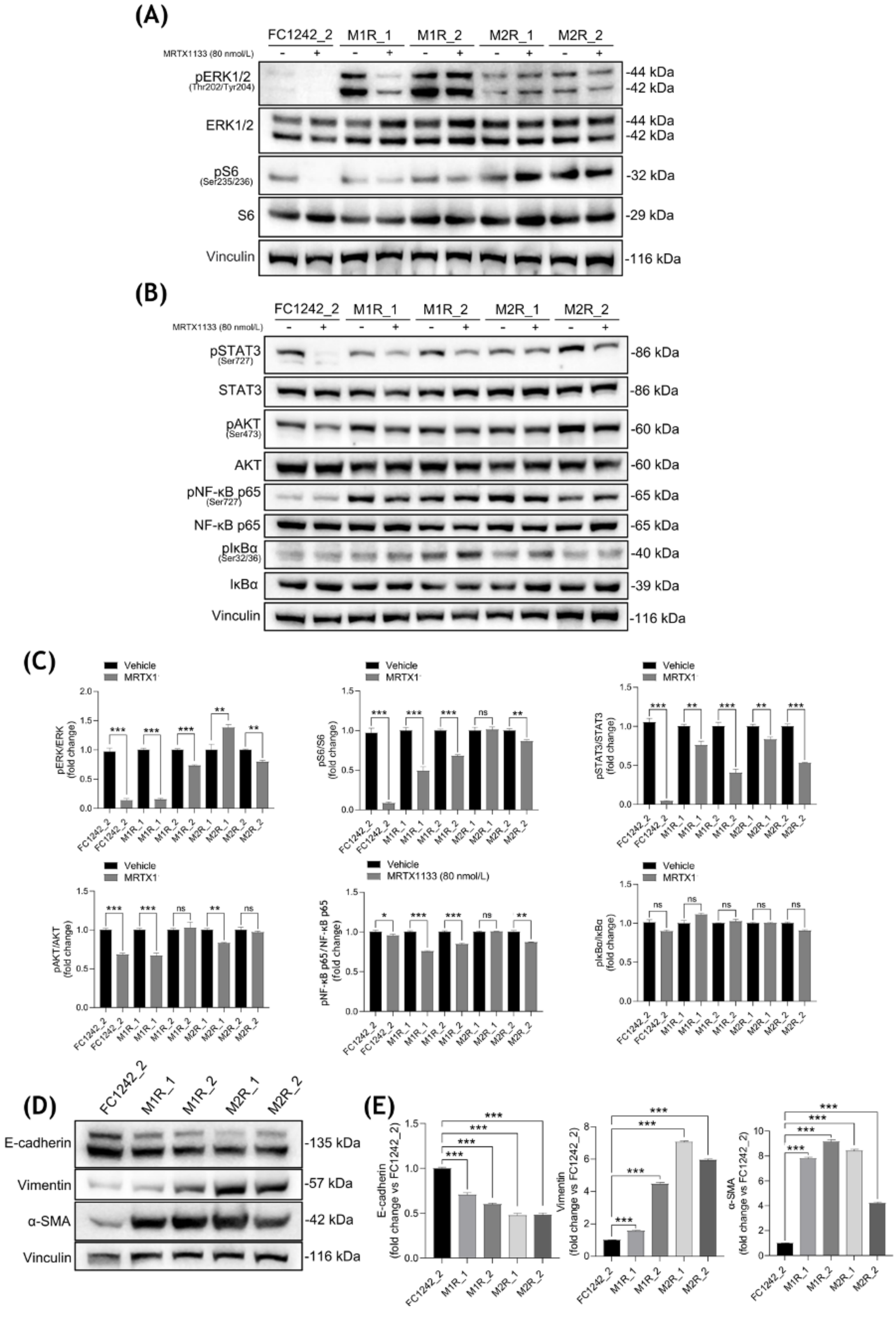

(A)
FC1242_2
M1R_1
M1R_2
M2R_1
M2R_2
MRTX1133 (80 nmol/L)
pERK1/2 (Thr202/Tyr204)
ERK1/2
pS6 (Ser235/236)
S6
Vinculin
-44 kDa
-42 kDa
-32 kDa
-29 kDa
-116 kDa
(B)
pSTAT3 (Ser727)
STAT3
pAKT (Ser473)
AKT
pNF-κB p65 (Ser727)
NF-κB p65
pIκBα (Ser32/36)
IκBα
Vinculin
-86 kDa
-60 kDa
-65 kDa
-40 kDa
-39 kDa
-116 kDa
(C)
Vehicle
MRTX1133 (80 nmol/L)
pERK/ERK (fold change)
pS6/S6 (fold change)
pSTAT3/STAT3 (fold change)
pAKT/AKT (fold change)
pNF-κB p65/NF-κB p65 (fold change)
pIκBα/IκBα (fold change)
(D)
E-cadherin
Vimentin
α-SMA
Vinculin
-135 kDa
-57 kDa
-42 kDa
-116 kDa
(E)
E-cadherin (fold change vs FC1242_2)
Vimentin (fold change vs FC1242_2)
α-SMA (fold change vs FC1242_2)

Supplementary Figure S4. *In vitro* models recapitulate MRTX1133 resistance in pancreatic cancer mouse model. (A) Western blotting analyses of downstream KRAS pathway activation: MAPK/ERK [pERK1/2 (Thr202/Tyr204) and ERK1/2] and PI3K/AKT/mTOR [pS6 (Ser235/236) and S6] signaling pathways. Non-resistant control and MRTX1133-resistant cell lines were treated with vehicle (–) or MRTX1133 at 80 nmol/L (+) for 6 hours, and analyzed for phosphorylation of ERK1/2 and S6. **(B)** Western blotting analyses of downstream KRAS pathway activation: JAK/STAT [pSTAT3 (Ser727) and STAT3], PI3K/AKT/mTOR [pAKT (Ser473) and AKT] and NF-κB [pNF-κB p65 (Ser727) and NF- κB p65, pIκBα (Ser32/36) and IκBα] signaling pathways. Non-resistant control and MRTX1133-resistant cell lines were treated with vehicle (–) or MRTX1133 at 80 nmol/L (+) for 6 hours, and analyzed for phosphorylation of STAT3, AKT, NF-κB p65 and IκBα. **(C)** Densitometric quantification of the Western blotting results shown in Supplementary Figures S4A and S4B. Bar graphs represent fold change relative to FC1242_2 vehicle-treated controls. **(D)** Western blotting analyses of EMT markers, including E-cadherin, Vimentin, and α-SMA, showing increased mesenchymal features in MRTX1133-resistant cells. **(E)** Densitometric quantification of the Western blotting results of EMT transition markers shown in Figure S4D. The concentration of 80 nmol/L MRTX1133 represents a biologically active yet submaximal inhibitory condition, allowing the assessment of cellular responses under partial pathway inhibition while preserving the viability of parental cells. Graphs (C and E) show the mean ± SD ($n = 3$). One-Way ANOVA was performed for statistical analyses (C and E), with $P < 0.05$ (*), $P < 0.01$ (**), and $P < 0.001$ (***). Abbreviations: AKT, Protein kinase B; ANOVA, analysis of variance; α-SMA, alpha–smooth muscle actin; E-cadherin, epithelial cadherin; EMT, epithelial-to-mesenchymal transition; ERK1/2, extracellular signal–regulated kinases 1 and 2; FC1242_2, parental MRTX1133-sensitive murine pancreatic cancer cell line; IκBα, inhibitor of kappa B alpha; JAK, Janus kinase; KRAS, Kirsten rat sarcoma viral oncogene

homolog; M1R, MRTX1133 1st resistance (first-generation MRTX1133-resistant cell line); M2R, MRTX1133 2nd resistance (second-generation MRTX1133-resistant cell line); MAPK, mitogen-activated protein kinase; MRTX1133, *KRAS*^{G12D} inhibitor; mTOR, mechanistic target of rapamycin; *n*, sample size; NF-κB, nuclear factor kappa-light-chain-enhancer of activated B cells; p, phosphorylated; *P*, probability value; PI3K, phosphoinositide 3-kinase; SD, standard deviation; Ser, serine; STAT3, signal transducer and activator of transcription 3; S6, ribosomal protein S6; Thr, threonine; Tyr, tyrosine.

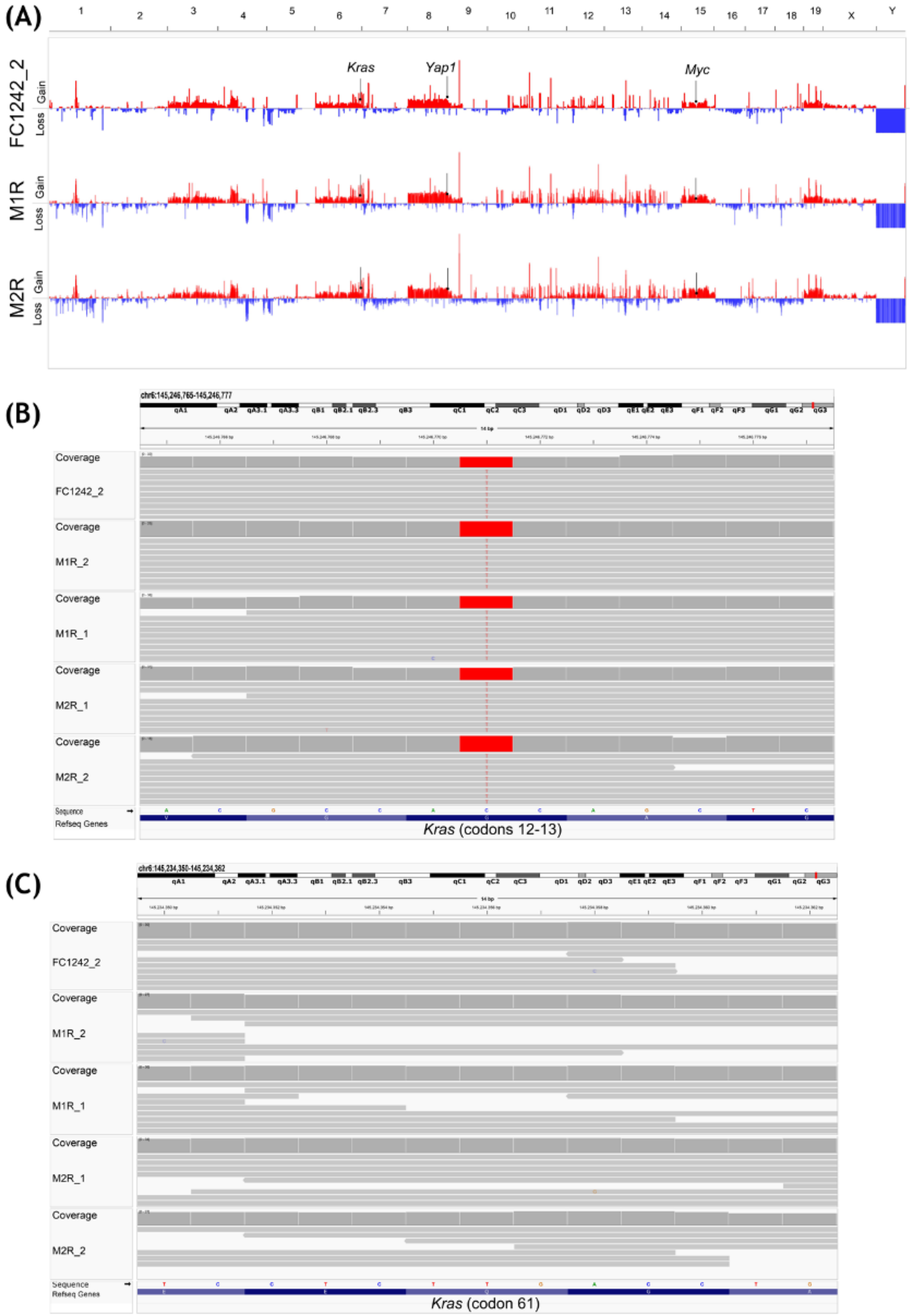

(A)
FC1242_2
M1R
M2R
Gain
Loss
Kras
Yap1
Myc
(B)
chr6:145,246,765-145,246,777
Coverage
FC1242_2
M1R_2
M1R_1
M2R_1
M2R_2
Sequence
Refseq Genes
Kras (codons 12-13)
(C)
chr6:145,234,350-145,234,362
Kras (codon 61)

Supplementary Figure S5. MRTX1133 resistance is not driven by secondary *Kras* mutations or CNV oncogene amplification. (A) Genome-wide CNV profiles of non-resistant control (FC1242_2) and MRTX1133-resistant lines (Biological replicates M1R_1 and M1R_2 are collapsed as M1R; M2R_1 and M2R_2 as M2R). CNV values are plotted along the chromosomes (top annotation) as log_2 ratios of copy number gains (red) and losses (blue). Annotated regions highlight known resistance-associated loci, including *Kras, Yap1, and Myc.* **(B-C)** Snapshot of *Kras* codons 12–13 (B) and codon 61 (C) in non-resistant control (FC1242_2) and MRTX1133-resistant lines (M1R_2, M1R_2, M2R_1, M2R_2), showing no evidence of *de novo* mutations. These specific hotspots were selected because they represent mutational regions with the highest frequency of oncogenic alteration in human cancer, including PDAC [12]. BAM coverage files are displayed with mismatches highlighted in red, while a G12D mutation is present in both the non-resistant control and MRTX1133-resistant, no additional mutations are observed at hotspot positions. Abbreviations: BAM, binary alignment map; CNV, copy number variation; FC1242_2, parental MRTX1133-sensitive murine pancreatic cancer cell line; G12D, glycine-to-aspartate substitution at codon 12; *Kras*, Kirsten rat sarcoma viral oncogene homolog; M1R, MRTX1133 1st resistance (first-generation MRTX1133-resistant cell line); M2R, MRTX1133 2nd resistance (second-generation MRTX1133-resistant cell line); *Myc*, myelocytomatosis oncogene; *Yap1*, yes-associated protein 1.

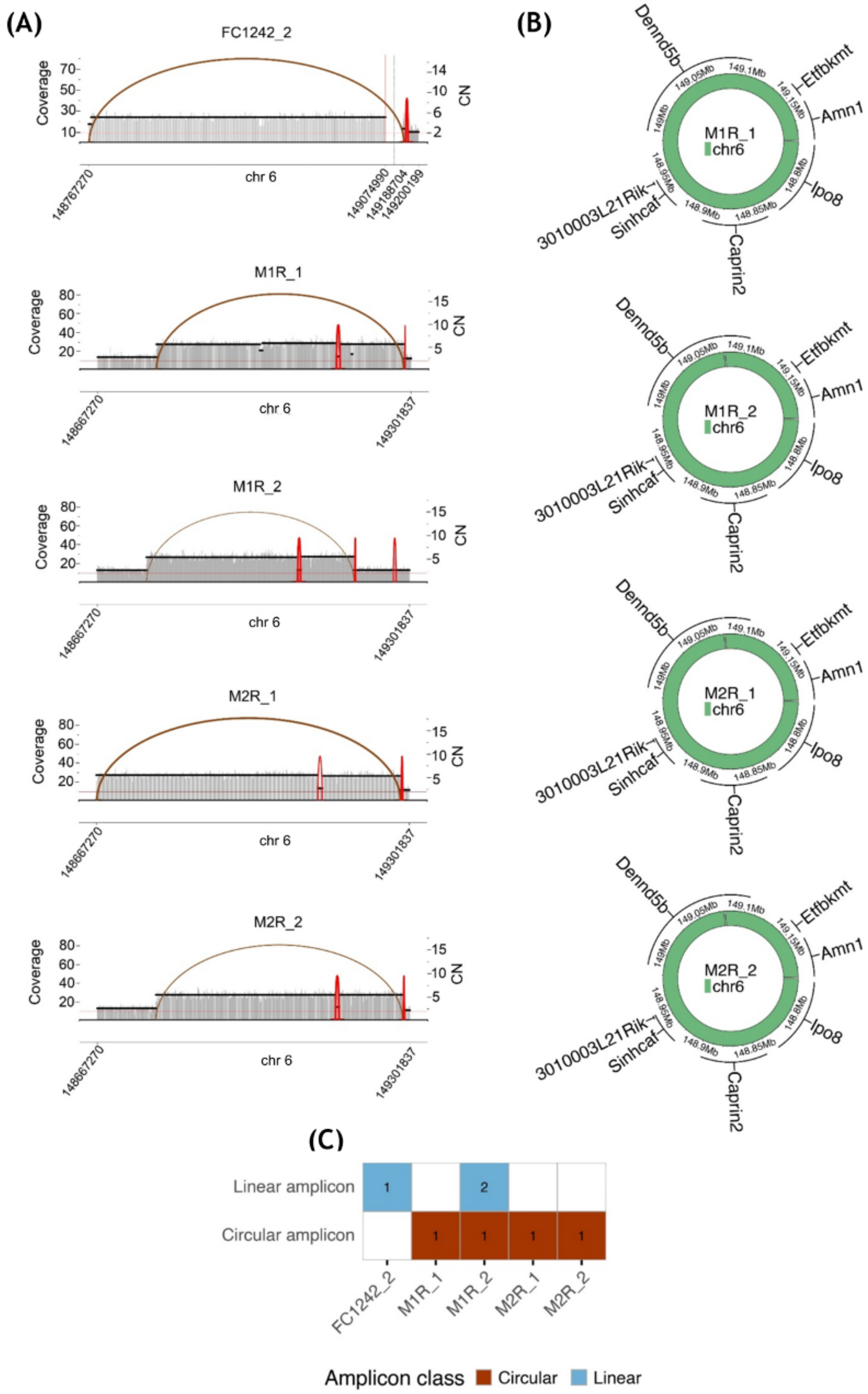

(A)
FC1242_2
Coverage
CN
chr 6
148767270
149074990
149188704
149200199
M1R_1
148667270
149301837
M1R_2
M2R_1
M2R_2
(B)
Dennd5b
Etfbkmt
Amn1
Ipo8
Caprin2
Sinhcaf
3010003L21Rik
M1R_1
M1R_2
M2R_1
M2R_2
chr6
(C)
Linear amplicon
Circular amplicon
FC1242_2
M1R_1
M1R_2
M2R_1
M2R_2
Amplicon class
Circular
Linear

Supplementary Figure S6. MRTX1133 resistance is not driven by ecDNA-mediated clonal evolution. (A) Structural variant view of AmpliconArchitect reconstructed amplicon structures in chromosome 6 for non-resistant control (FC1242_2) and MRTX1133-resistant cell lines (M1R_1, M1R_2, M2R_1, M2R_2). Structural variant view shows the coverage depth on the left, and the copy number on the right. Discordant genomic connections are represented by curves spanning copy number segments (brown = “duplication-like”, red = “deletion-like”). **(B)** Representative Circos plots showing the reconstructed structure of the circular amplicons identified by AmpliconArchitect in the MRTX1133-resistant samples (M1R_1, M1R_2, M2R_1, M2R_2). The green bars represent the genomic segments that compose the circular structure, while the outer annotation indicates the main genes located on the amplicon and their relative length along the circular structure (black line). **(C)** Table reporting the number of linear and circular amplicons for each sample, obtained by AmpliconClassifier. Abbreviations: 3010003L21Rik, RIKEN cDNA 3010003L21 gene; Amn1, antagonist of mitotic exit network 1 homolog; Caprin2, caprin family member 2; chr, chromosome; CN, copy number; Dennd5B, DENN Domain Containing 5B; ecDNA, extrachromosomal DNA; Etfbkmt, electron transfer flavoprotein subunit beta lysine methyltransferase; FC1242_2, parental MRTX1133-sensitive murine pancreatic cancer cell line; Ipo8, importin 8; M1R, MRTX1133 1st resistance (first-generation MRTX1133-resistant cell line); M2R, MRTX1133 2nd resistance (second-generation MRTX1133-resistant cell line); Sinhcaf, SIN3-HDAC complex associated factor.

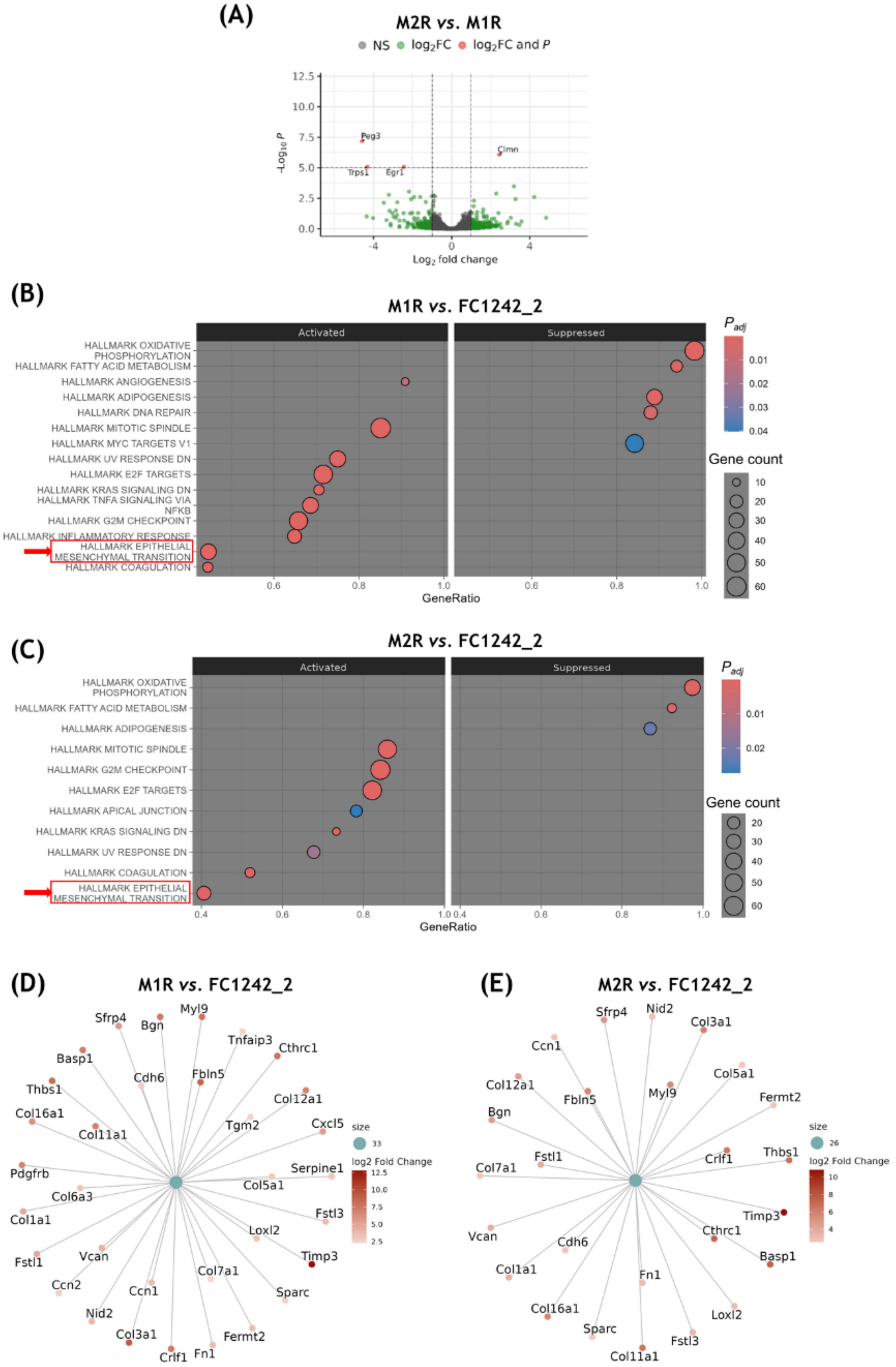
(A)
M2R vs. M1R
NS
log2FC
log2FC and P
Peg3
Trps1
Egr1
Clmn
Log2 fold change
-Log10 P
(B)
M1R vs. FC1242_2
Activated
Suppressed
HALLMARK OXIDATIVE PHOSPHORYLATION
HALLMARK FATTY ACID METABOLISM
HALLMARK ANGIOGENESIS
HALLMARK ADIPOGENESIS
HALLMARK DNA REPAIR
HALLMARK MITOTIC SPINDLE
HALLMARK MYC TARGETS V1
HALLMARK UV RESPONSE DN
HALLMARK E2F TARGETS
HALLMARK KRAS SIGNALING DN
HALLMARK TNFA SIGNALING VIA NFKB
HALLMARK G2M CHECKPOINT
HALLMARK INFLAMMATORY RESPONSE
HALLMARK EPITHELIAL MESENCHYMAL TRANSITION
HALLMARK COAGULATION
GeneRatio
Padj
Gene count
(C)
M2R vs. FC1242_2
Activated
Suppressed
HALLMARK OXIDATIVE PHOSPHORYLATION
HALLMARK FATTY ACID METABOLISM
HALLMARK ADIPOGENESIS
HALLMARK MITOTIC SPINDLE
HALLMARK G2M CHECKPOINT
HALLMARK E2F TARGETS
HALLMARK APICAL JUNCTION
HALLMARK KRAS SIGNALING DN
HALLMARK UV RESPONSE DN
HALLMARK COAGULATION
HALLMARK EPITHELIAL MESENCHYMAL TRANSITION
GeneRatio
Padj
Gene count
(D)
M1R vs. FC1242_2
size
log2 Fold Change
Sfrp4 Bgn Myl9 Tnfaip3 Cthrc1 Basp1 Cdh6 Fbln5 Col12a1 Thbs1 Col16a1 Col11a1 Tgm2 Cxcl5 Serpine1 Pdgfrb Col5a1 Col6a3 Col1a1 Fstl3 Loxl2 Timp3 Fstl1 Vcan Col7a1 Ccn2 Ccn1 Sparc Nid2 Col3a1 Fermt2 Crlf1 Fn1
(E)
M2R vs. FC1242_2
size
log2 Fold Change
Sfrp4 Nid2 Col3a1 Ccn1 Col5a1 Col12a1 Fbln5 Myl9 Fermt2 Bgn Fstl1 Crlf1 Thbs1 Col7a1 Timp3 Cthrc1 Vcan Cdh6 Basp1 Col1a1 Fn1 Col16a1 Loxl2 Sparc Fstl3 Col11a1

Supplementary Figure S7. Transcriptomic profiling reveals EMT-related transcriptional pathways upregulated in MRTX1133-resistant pancreatic cancer cells. (A) Volcano plot showing differentially expressed genes in M2R (integrated data with M2R_1 and M2R_2) cell lines compared to M1R (integrated data with M1R_1 and M1R_2). Significance thresholds were set at $|\log_2\text{fold change}| > 1$, and $-\log_{10}P > 5$ **(B-C)** GSEA plots showing the pathways enriched based on the differentially expressed genes from the M1R (integrated data with M1R_1 and M1R_2) *vs.* FC1242_2 non-resistant control cells (B) and M2R (integrated data with M2R_1 and M2R_2) *vs.* FC1242_2 non-resistant control cells (C). The MSigDB Hallmark gene set was used for GSEA. **(D)** EMT-related gene network plot for M1R cells, highlighting genes contributing to enrichment of the HALLMARK_EPITHELIAL_MESENCHYMAL_TRANSITION pathway. Node color indicates $\log_2$ fold change, and node size reflects contribution to enrichment score. **(E)** EMT-related gene network plot for M2R cells, highlighting genes contributing to enrichment of the HALLMARK_EPITHELIAL_MESENCHYMAL_TRANSITION pathway. Node color indicates $\log_2$ fold change, and node size reflects contribution to enrichment score. Abbreviations: EMT, epithelial-to-mesenchymal transition; FC, fold change; FC1242_2, parental MRTX1133-sensitive murine pancreatic cancer cell line; GSEA, gene set enrichment analysis; M1R, MRTX1133 1st resistance (first-generation MRTX1133-resistant cell line); M2R, MRTX1133 2nd resistance (second-generation MRTX1133-resistant cell line); ns, not significant; *P*, probability value.

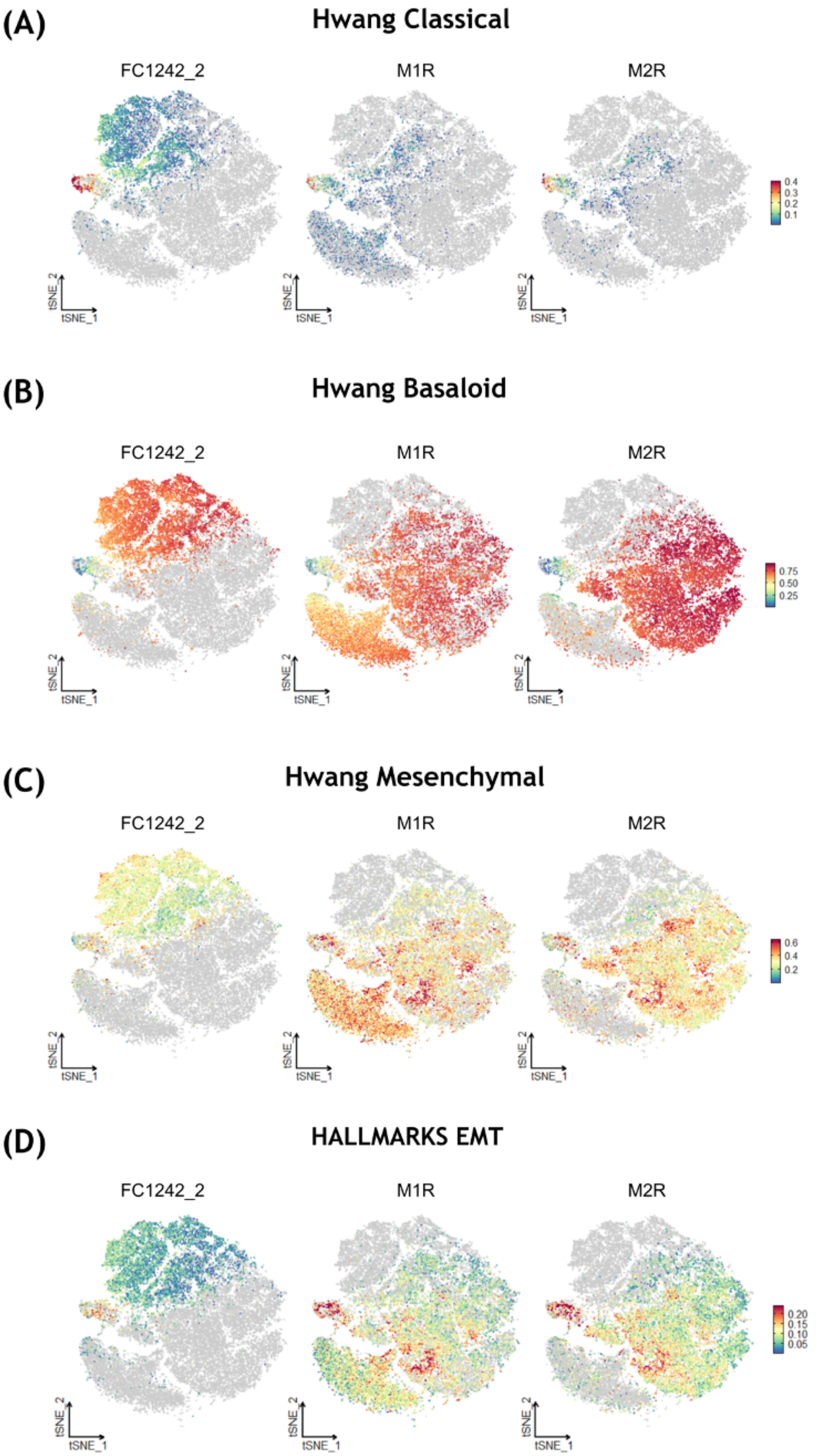
(A)
Hwang Classical
FC1242_2
M1R
M2R
0.4
0.3
0.2
0.1
tSNE_2
tSNE_1
(B)
Hwang Basaloid
FC1242_2
M1R
M2R
0.75
0.50
0.25
tSNE_2
tSNE_1
(C)
Hwang Mesenchymal
FC1242_2
M1R
M2R
0.6
0.4
0.2
tSNE_2
tSNE_1
(D)
HALLMARKS EMT
FC1242_2
M1R
M2R
0.20
0.15
0.10
0.05
tSNE_2
tSNE_1

Supplementary Figure S8. MRTX1133-resistant cells exhibit transcriptional heterogeneity and subtype signatures at single-cell resolution, highlighting the acquisition of plasticity and a mesenchymal phenotype. (A-C) Module scoring for Hwang single cell molecular signature [10], a curated gene sets derived from single-cell RNA-sequencing data representing transcriptional programs associated with distinct molecular phenotypes, across various cell lines (FC1242_2, M1R, M2R): Classical (A), Basaloid (B), Mesenchymal (C). **(D)** Module scoring for HALLMARK EMT signature (https://www.gsea-msigdb.org/gsea/msigdb/mouse/geneset/HALLMARK_EPITHELIAL_MESENCHYMAL_TRANSITION.html) across various cell lines. M1R was the integrated data from M1R_1 and M1R_2, M2R was the integrated data from M2R_1 and M2R_2. Abbreviations: EMT, epithelial-to-mesenchymal transition; FC1242_2, parental MRTX1133-sensitive murine pancreatic cancer cell line; M1R, MRTX1133 1st resistance (first-generation MRTX1133-resistant cell line); M2R, MRTX1133 2nd resistance (second-generation MRTX1133-resistant cell line).

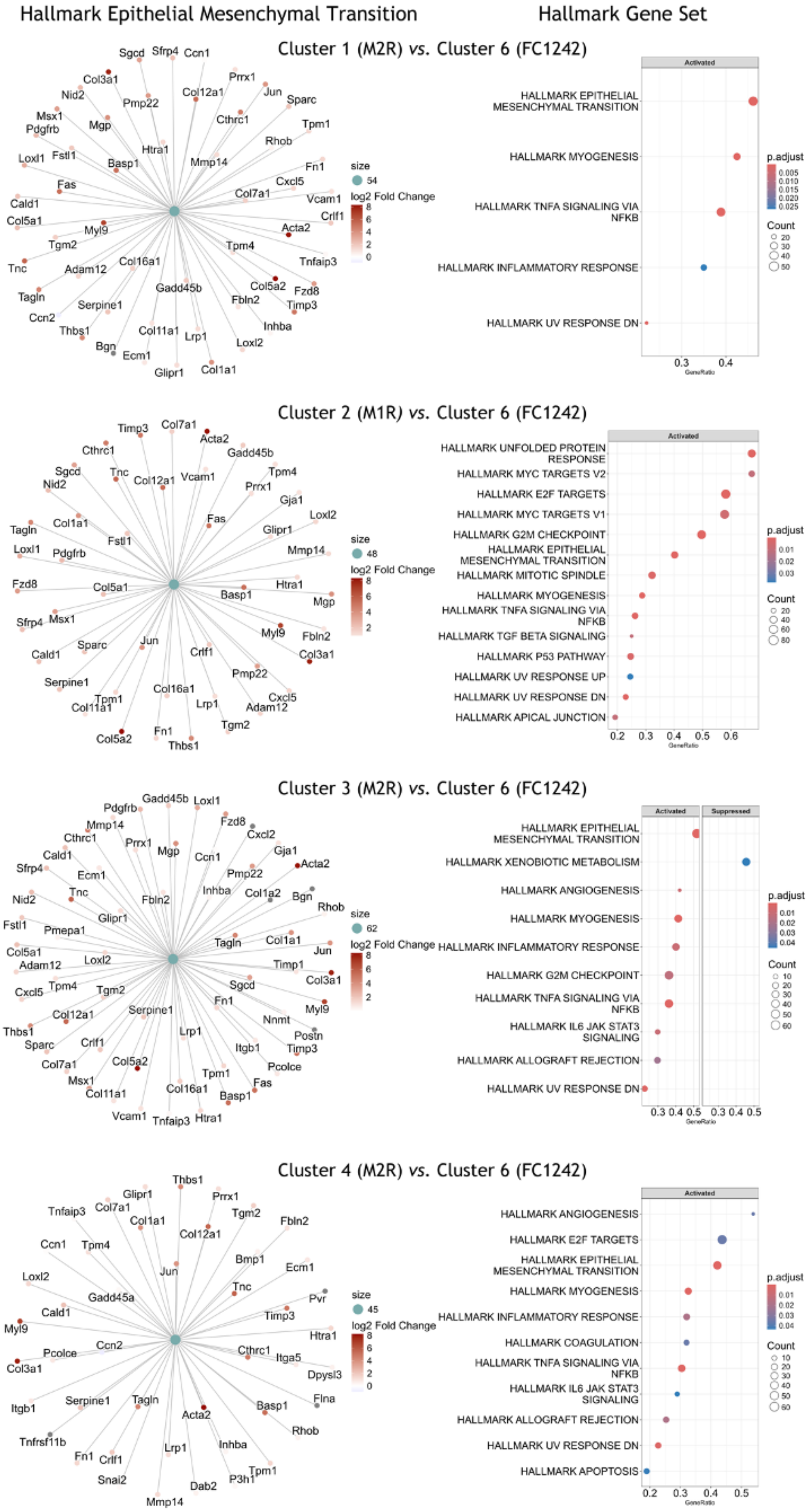

Hallmark Epithelial Mesenchymal Transition
Hallmark Gene Set
Cluster 1 (M2R) vs. Cluster 6 (FC1242)
size
log2 Fold Change
Activated
HALLMARK EPITHELIAL MESENCHYMAL TRANSITION
HALLMARK MYOGENESIS
HALLMARK TNFA SIGNALING VIA NFKB
HALLMARK INFLAMMATORY RESPONSE
HALLMARK UV RESPONSE DN
p.adjust
Count
Cluster 2 (M1R) vs. Cluster 6 (FC1242)
HALLMARK UNFOLDED PROTEIN RESPONSE
HALLMARK MYC TARGETS V2
HALLMARK E2F TARGETS
HALLMARK MYC TARGETS V1
HALLMARK G2M CHECKPOINT
HALLMARK EPITHELIAL MESENCHYMAL TRANSITION
HALLMARK MITOTIC SPINDLE
HALLMARK MYOGENESIS
HALLMARK TNFA SIGNALING VIA NFKB
HALLMARK TGF BETA SIGNALING
HALLMARK P53 PATHWAY
HALLMARK UV RESPONSE UP
HALLMARK UV RESPONSE DN
HALLMARK APICAL JUNCTION
Cluster 3 (M2R) vs. Cluster 6 (FC1242)
Activated
Suppressed
HALLMARK EPITHELIAL MESENCHYMAL TRANSITION
HALLMARK XENOBIOTIC METABOLISM
HALLMARK ANGIOGENESIS
HALLMARK MYOGENESIS
HALLMARK INFLAMMATORY RESPONSE
HALLMARK G2M CHECKPOINT
HALLMARK TNFA SIGNALING VIA NFKB
HALLMARK IL6 JAK STAT3 SIGNALING
HALLMARK ALLOGRAFT REJECTION
HALLMARK UV RESPONSE DN
Cluster 4 (M2R) vs. Cluster 6 (FC1242)
HALLMARK ANGIOGENESIS
HALLMARK E2F TARGETS
HALLMARK EPITHELIAL MESENCHYMAL TRANSITION
HALLMARK MYOGENESIS
HALLMARK INFLAMMATORY RESPONSE
HALLMARK COAGULATION
HALLMARK TNFA SIGNALING VIA NFKB
HALLMARK IL6 JAK STAT3 SIGNALING
HALLMARK ALLOGRAFT REJECTION
HALLMARK UV RESPONSE DN
HALLMARK APOPTOSIS

Supplementary Figure S9. The transcriptomic landscape of the MRTX1133-resistant clusters highlights the overexpression of cancer hallmarks associated with an acquired mesenchymal program. (A-D) EMT-related gene network and GSEA plots comparing Seurat clusters enriched in MRTX1133-resistant cell lines with the non-resistant control FC1242_2 cell line. Specifically, cluster 2 (M1R) to cluster 6 (FC1242_2) (A), cluster 1 (M2R) to cluster 6 (FC1242_2) (B), cluster 3 (M2R) to cluster 6 (FC1242_2) (C), and cluster 4 (M2R) to cluster 6 (FC1242_2) (D). The plots highlighted the genes contributing to enrichment of the HALLMARK_EPITHELIAL_MESENCHYMAL_TRANSITION pathway. Node color indicates log_2 fold change, and node size reflects contribution to enrichment score. Abbreviations: EMT, epithelial-to-mesenchymal transition; FC1242_2, parental MRTX1133-sensitive murine pancreatic cancer cell line; GSEA, gene set enrichment analysis; M1R, MRTX1133 1st resistance (first-generation MRTX1133-resistant cell line); M2R, MRTX1133 2nd resistance (second-generation MRTX1133-resistant cell line).

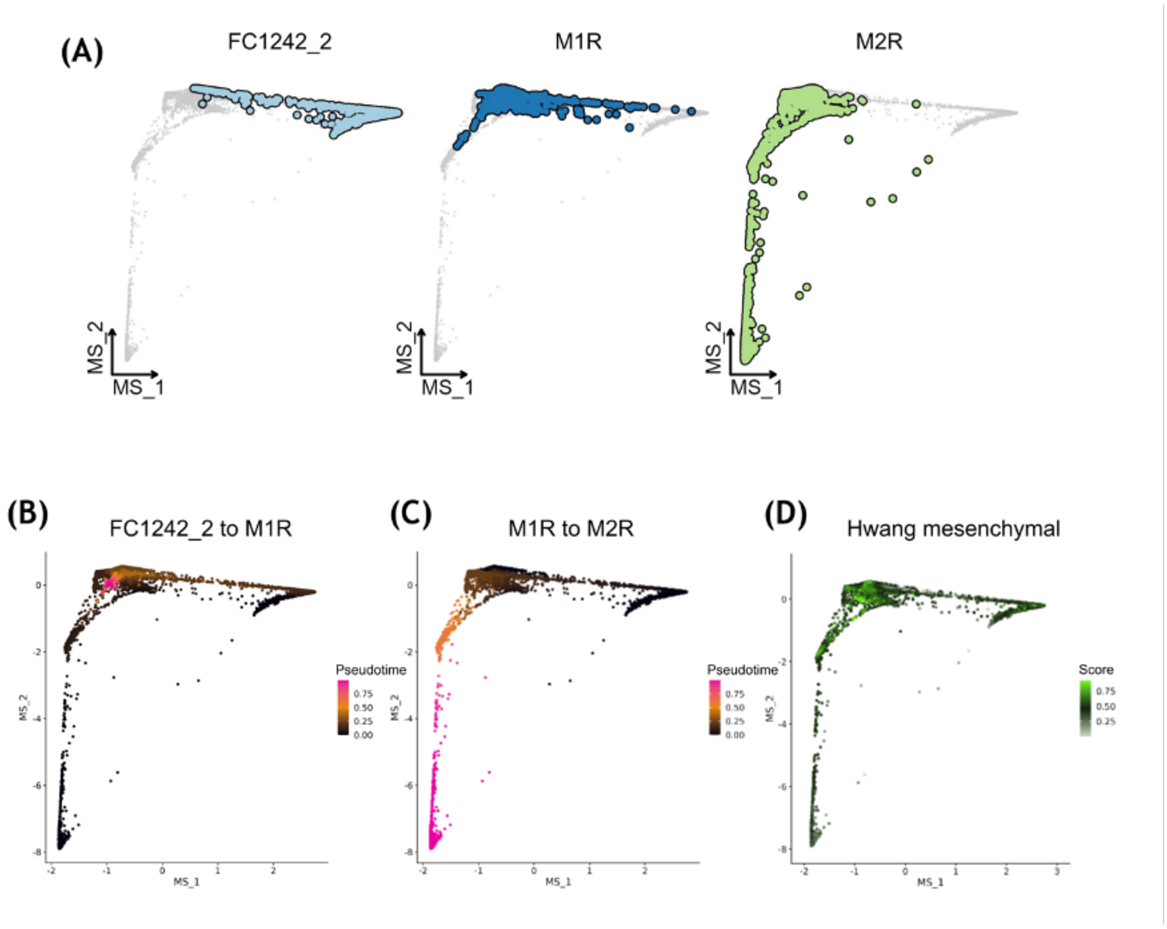

Supplementary Figure S10. MRTX1133-resistant cell lines associate with a more mesenchymal and plastic phenotype. (A) Palantir MS dimension reduction showing the plasticity of the different cell lines (FC1242_2, M1R and M2R). **(B-C)** Cell fate plots based on Palantir algorithm: from FC1242_2 to M1R (B), and from M1R to M2R (C). FC1242_2 cells were used as the starting point (pseudotime = 0), and the increase in pseudotime reflects the progression toward resistant states. **(D)** Palantir MS dimension reduction showing the module score calculated for the Hwang mesenchymal signature; the score appears to be associated with the plasticity of clusters 2, 3 and 4 characteristics of MRTX1133-resistant cell. Abbreviations: FC1242_2, parental MRTX1133-sensitive murine pancreatic cancer cell line; MS, multiscale space; M1R, MRTX1133 1st resistance (first-generation MRTX1133-resistant cell line); M2R, MRTX1133 2nd resistance (second-generation MRTX1133-resistant cell line).

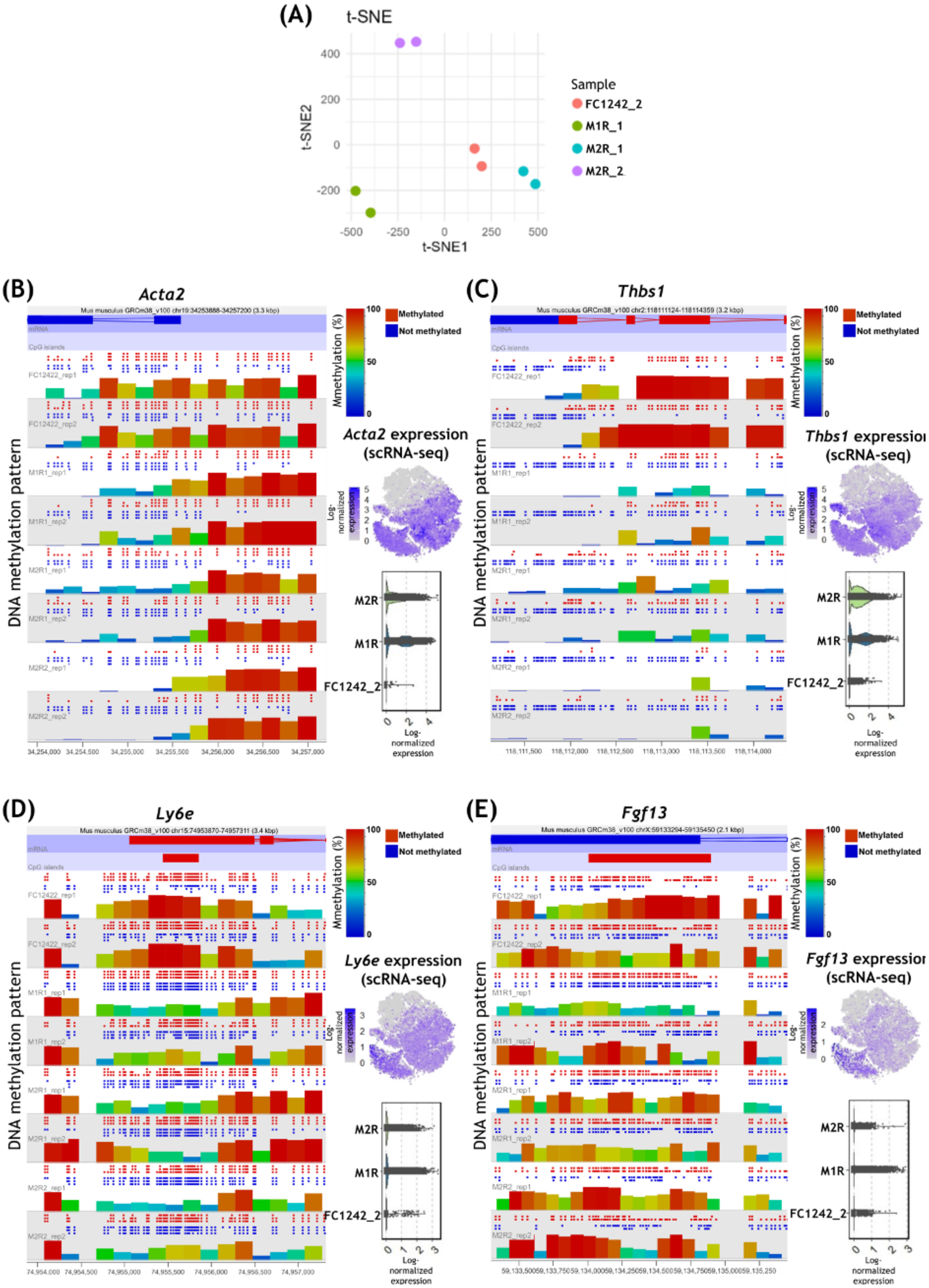

(A)
t-SNE
t-SNE1
t-SNE2
Sample
FC1242_2
M1R_1
M2R_1
M2R_2
(B)
Acta2
Acta2 expression (scRNA-seq)
Methylated
Not methylated
DNA methylation pattern
(C)
Thbs1
(D)
Ly6e
Ly6e expression (scRNA-seq)
(E)
Fgf13
M2R
M1R
FC1242_2
Log-normalized expression

Supplementary Figure S11. Epigenetic reprogramming promotes a mesenchymal phenotype shift in MRTX1133-resistant cell lines. (A) PCA of DNA methylation profiles shows separation of cell lines (FC1242_2, M1R_1, M2R_1, M2R_2). Each point represents a technical replicate. **(B-E)** Demethylation profiles of EMT-related genes that may contribute to the acquisition of a mesenchymal phenotype, in particular *Acta2* (B), *Thbs1* (C), *Ly6e* (D) and *Fgf13* (E). For each gene, DNA methylation patterns, single-cell expression across samples (t-SNE plots), and violin plots representing gene expression in the indicated cell lines (FC1242_2, M1R (integrated data with M1R_1 and M1R_2), M2R (integrated data with M2R_1 and M2R_2)) are shown. Abbreviations: *Acta2*, alpha smooth muscle actin; EMT, epithelial-to-mesenchymal transition; FC1242_2, parental MRTX1133-sensitive murine pancreatic cancer cell line; *Fgf13*, fibroblast growth factor 13; *Ly6e*, lymphocyte antigen 6 complex locus E; M1R, MRTX1133 1st resistance (first-generation MRTX1133-resistant cell line); M2R, MRTX1133 2nd resistance (second-generation MRTX1133-resistant cell line); PCA, principal component analysis; *Thbs1*, thrombospondin 1; t-SNE, t-distributed stochastic neighbor embedding.

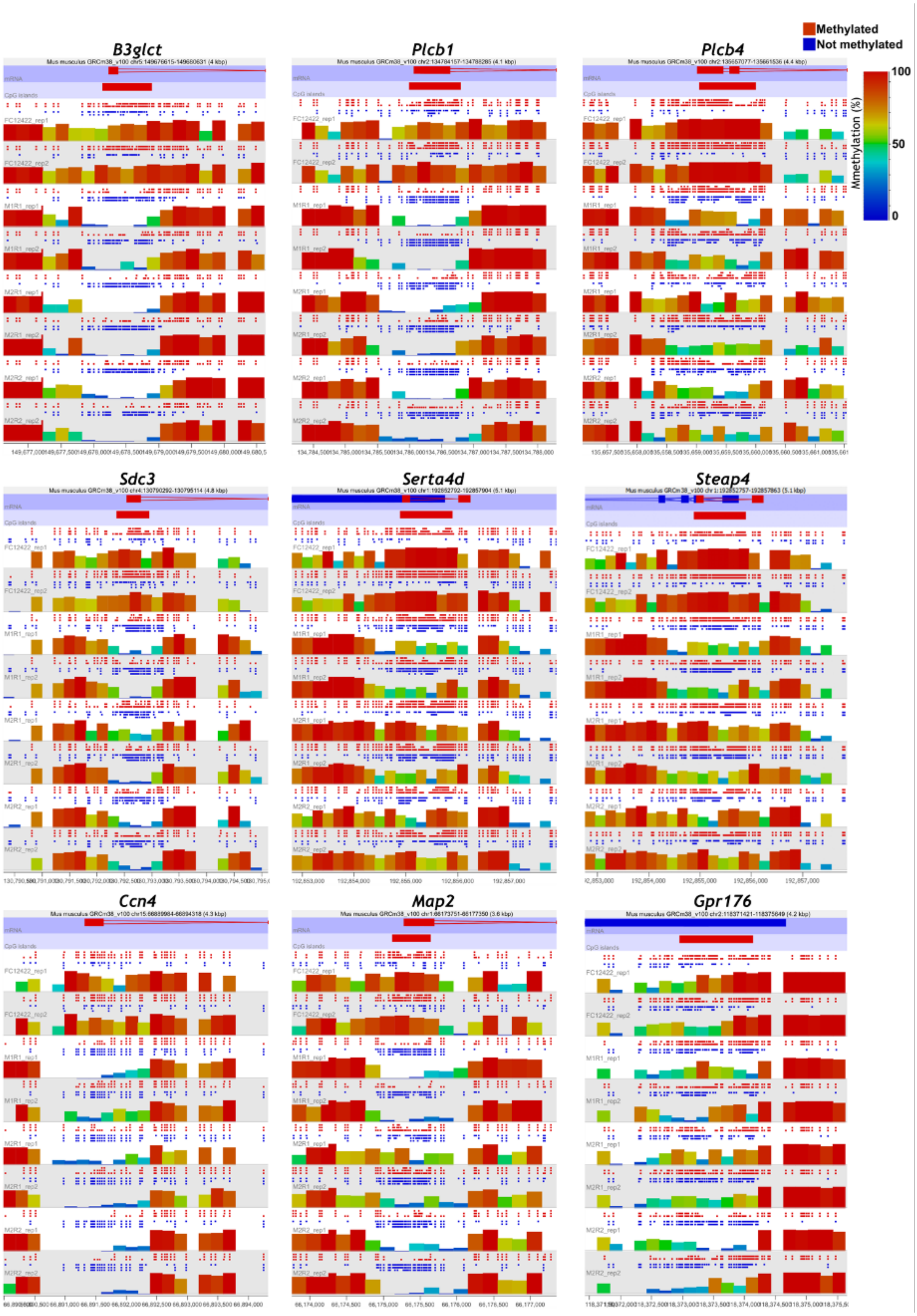

Methylated
Not methylated
Mmethylation (%)
100
50
0
B3glct
Plcb1
Plcb4
Sdc3
Serta4d
Steap4
Ccn4
Map2
Gpr176
FC12422_rep1
FC12422_rep2
M1R1_rep1
M1R1_rep2
M2R1_rep1
M2R1_rep2
M2R2_rep1
M2R2_rep2

Supplementary Figure S12. MRTX1133-resistant cell lines display a consistent pattern of demethylation at genes associated with EMT transition. DNA methylation profiles of additional EMT-related genes that may contribute to the acquisition of a mesenchymal phenotype in MRTX1133-resistant cell lines: *B3glct, Plcb1, Plcb4, Sdc3, Serta4d, Steap4, Ccn4, Map2, and Gpr176.* Abbreviations: *B3glct*, beta-1,3-glucosyltransferase; *Ccn4*, cellular communication network factor 4 (also known as WISP1); EMT, epithelial-to-mesenchymal transition; *Gpr176*, G protein-coupled receptor 176; *Map2*, microtubule-associated protein 2; *Plcb1*, phospholipase C beta 1; *Plcb4*, phospholipase C beta 4; *Sdc3*, syndecan 3; *Sertad4*, SERTA domain-containing protein 4; *Steap4*, six transmembrane epithelial antigen of prostate 4.

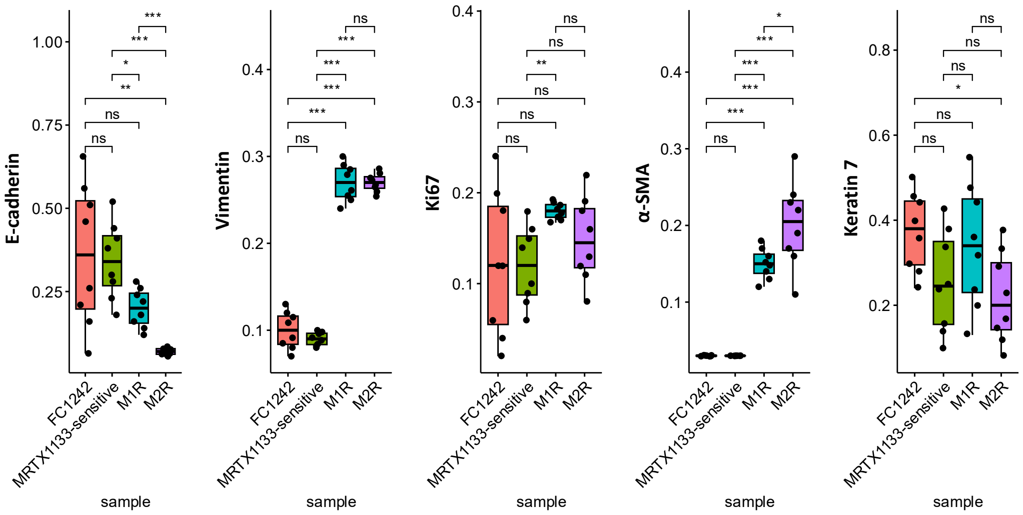

Supplementary Figure S13. Semi-quantitative analysis of IHC staining of Figure 1Q revealed a phenotypic shift toward a more mesenchymal state in MRTX1133-resistant tumors. Values represent the fraction of DAB-positive area for each marker across the indicated conditions (Vehicle, MRTX1133-sensitive, M1R, and M2R; n = 8 each group). The data show a progressive reduction of epithelial features (E-cadherin) and a concomitant increase in mesenchymal markers (Vimentin and α-SMA) in resistant tumors, consistent with a shift toward a mesenchymal phenotype. Ki67 levels remain comparable across conditions, in line with the absence of a proliferative advantage. Abbreviations: α-SMA, Alpha smooth muscle actin; Ki67, proliferation marker; M1R, MRTX1133 1st resistance (first-generation MRTX1133-resistant cell line); M2R, MRTX1133 2nd resistance (second-generation MRTX1133-resistant cell line); MRTX1133, *KRAS*^{G12D} inhibitor; ns, not significant.

Supplementary Tables

Supplementary Table S1. Amplicon classifier results summary.

Sample name	AA amplicon number	Feature ID	Classification	Amplicon type	Location	All genes	Complexity score	Captured interval length	Feature median copy number	Feature maximum copy number
FC1242_2	2	DNA_1_amplicon2_Linear_1	Linear	Linear	'chr6:148767549-149074778'	'3010003L21Rik', 'Caprin2', 'Fam60a', 'Gm15779', 'Gm15780', 'Gm15781', 'Gm22578', 'Gm23462', 'Gm25539', 'Gm26540', 'Gm43908', 'Gm43913', 'Gm44143', 'Gm7618', 'Ipo8', 'RP23-355N5.6'	0.919	307,229	4.966	4.966
M1R_2	2	DNA_2_amplicon2_Linear_1	Linear	Linear	'chr4:43236891-44731200', 'chr4:44880001-46579900'	'1700055D18Rik', '4930517J16Rik', '5430416O09Rik', '5730488B01Rik', 'Aldh1b1', 'Anp32b', 'Arhgef39', 'Atp8b5', 'Car9', 'Ccdc107', 'Ccdc180', 'Ccin', 'Cd72', 'Clta', 'Creb3', 'Dcaf10', 'Exosc3', 'Fam166b', 'Fam221b', 'Fbxo10', 'Foxe1', 'Frmpd1', 'Frmpd1os', 'Gba2', 'Glipr2', 'Gm12408', 'Gm12409', 'Gm12410', 'Gm12411', 'Gm12412', 'Gm12443', 'Gm12444', 'Gm12445', 'Gm12446', 'Gm12447', 'Gm12454', 'Gm12455', 'Gm12462', 'Gm12472',	0.510	3,194,208	5.002	5.044

						'Gm12473', 'Gm12481', 'Gm12492', 'Gm12493', 'Gm12503', 'Gm12504', 'Gm12566', 'Gm12585', 'Gm12677', 'Gm12678', 'Gm12679', 'Gm16731', 'Gm22055', 'Gm22247', 'Gm22518', 'Gm22706', 'Gm23250', 'Gm23257', 'Gm24376', 'Gm25262', 'Gm25692', 'Gm27265', 'Gm829', 'Gne', 'Grhpr', 'Hemgn', 'Hint2', 'Hrct1', 'Igfbpl1', 'Melk', 'Mir5106', 'Mir5120', 'Msmp', 'Nans', 'Ncbp1', 'Npr2', 'Olfr155', 'Olfr156', 'Olfr157', 'Olfr159', 'Olfr269-ps1', 'Olfr29-ps1', 'Olfr70', 'Olfr71', 'Pax5', 'Polr1e', 'Reck', 'Rgp1', 'Rmrp', 'Rnf38', 'Rusc2', 'Shb', 'Sit1', 'Slc25a51', 'Spag8', 'Stra6l', 'Tdrd7', 'Tesk1', 'Tln1', 'Tmem8b', 'Tmod1', 'Tomm5', 'Tpm2', 'Trim14', 'Trmo', 'Trmt10b', 'Tstd2', 'Xpa', 'Zbtb5'				
M1R_2	3	DNA_2_amplicon3_Linear_1	Linear	Linear	'chr4:47582033-47780552'	'Gm27601', 'Gm27668'	1.151	198,519	4.767	4.767
M1R_2	4	DNA_2_amplicon4_ecDNA_1	ecDNA	Circular	'chr6:148767642-149074778', 'chr6:149080647-149189453'	'3010003L21Rik', 'Amn1', 'Caprin2', 'Dennd5b', 'Fam60a', 'Gm10203',	0.693	415,942	5.652	5.652

						'Gm15779', 'Gm15780', 'Gm15781', 'Gm22578', 'Gm23462', 'Gm25539', 'Gm26540', 'Gm43908', 'Gm43913', 'Gm44143', 'Gm7618', 'Ipo8', 'Mettl20', 'RP23-355N5.6'				
M1R_1	2	DNA_4_amplicon2_ecDNA_1	ecDNA	Circular	'chr6:148767527-149074780', 'chr6:149080648-149189453'	'3010003L21Rik', 'Amn1', 'Caprin2', 'Dennd5b', 'Fam60a', 'Gm10203', 'Gm15779', 'Gm15780', 'Gm15781', 'Gm22578', 'Gm23462', 'Gm25539', 'Gm26540', 'Gm43908', 'Gm43913', 'Gm44143', 'Gm7618', 'Ipo8', 'Mettl20', 'RP23-355N5.6'	0.693	416,058	5.684	5.684
M2R_1	2	DNA_5_amplicon2_ecDNA_1	ecDNA	Circular	'chr6:148767557-149074778', 'chr6:149080647-149189453'	'3010003L21Rik', 'Amn1', 'Caprin2', 'Dennd5b', 'Fam60a', 'Gm10203', 'Gm15779', 'Gm15780', 'Gm15781', 'Gm22578', 'Gm23462', 'Gm25539', 'Gm26540', 'Gm43908', 'Gm43913', 'Gm44143', 'Gm7618', 'Ipo8', 'Mettl20', 'RP23-355N5.6'	0.689	416,027	5.624	5.624
M2R_2	2	DNA_6_amplicon2_ecDNA_1	ecDNA	Circular	'chr6:148767527-149074778', 'chr6:149080647-149189453'	'3010003L21Rik', 'Amn1', 'Caprin2', 'Dennd5b', 'Fam60a', 'Gm10203', 'Gm15779',	0.693	416,057	5.615	5.615

						'Gm15780', 'Gm15781', 'Gm22578', 'Gm23462', 'Gm25539', 'Gm26540', 'Gm43908', 'Gm43913', 'Gm44143', 'Gm7618', 'Ipo8', 'Mettl20', 'RP23-355N5.6'				

Abbreviations: chr, chromosome; ecDNA, extrachromosomal DNA; FC1242_2, parental MRTX1133-sensitive murine pancreatic cancer cell line; M1R, MRTX1133 1st resistance (first-generation MRTX1133-resistant cell line); M2R, MRTX1133 2nd resistance (second-generation MRTX1133-resistant cell line).
